# Reduced Mrp2 surface availability as PI3Kγ-mediated hepatocytic dysfunction reflecting a hallmark of cholestasis in sepsis

**DOI:** 10.1038/s41598-020-69901-3

**Published:** 2020-08-04

**Authors:** Anne J. Beer, David Hertz, Eric Seemann, Martina Beretta, Martin Westermann, Reinhard Bauer, Michael Bauer, Michael M. Kessels, Britta Qualmann

**Affiliations:** 1 Institute of Biochemistry I, Jena University Hospital, Friedrich Schiller University Jena, 07743 Jena, Germany; 2 Center for Sepsis Control and Care and Department of Anesthesiology and Intensive Care Medicine, Jena University Hospital, Friedrich Schiller University Jena, Erlanger Allee 101, 07747 Jena, Germany; 3 Institute of Molecular Cell Biology, CMB-Center for Molecular Biomedicine, Jena University Hospital, Friedrich Schiller University, Hans-Knöll-Straße 2, 07745 Jena, Germany; 4 Electron Microscopy Center, Jena University Hospital, Friedrich Schiller University Jena, Ziegelmühlenweg 1, 07743 Jena, Germany; 5 Present Address: Research Center Borstel, Leibniz Lung Center, Priority Area Infections, Parkallee 1-40, 23845 Borstel, Germany; 6 Present Address: School of Biotechnology and Biomolecular Sciences, University of New South Wales Sydney, Sydney, Australia

**Keywords:** Cell biology, Molecular biology, Physiology, Zoology, Diseases

## Abstract

Sepsis-associated liver dysfunction manifesting as cholestasis is common during multiple organ failure. Three hepatocytic dysfunctions are considered as major hallmarks of cholestasis in sepsis: impairments of microvilli covering canalicular membranes, disruptions of tight junctions sealing bile-collecting canaliculae and disruptions of Mrp2-mediated hepatobiliary transport. PI3Kγ loss-of-function was suggested as beneficial in early sepsis. Yet, the PI3Kγ-regulated cellular processes in hepatocytes remained largely unclear. We analysed all three sepsis hallmarks for responsiveness to massive PI3K/Akt signalling and PI3Kγ loss-of-function, respectively. Surprisingly, neither microvilli nor tight junctions were strongly modulated, as shown by electron microscopical studies of mouse liver samples. Instead, quantitative electron microscopy proved that solely Mrp2 surface availability, i.e. the third hallmark, responded strongly to PI3K/Akt signalling. Mrp2 plasma membrane levels were massively reduced upon PI3K/Akt signalling. Importantly, Mrp2 levels at the plasma membrane of PI3Kγ KO hepatocytes remained unaffected upon PI3K/Akt signalling stimulation. The effect explicitly relied on PI3Kγ’s enzymatic ability, as shown by PI3Kγ kinase-dead mice. Keeping the surface availability of the biliary transporter Mrp2 therefore is a cell biological process that may underlie the observation that PI3Kγ loss-of-function protects from hepatic excretory dysfunction during early sepsis and Mrp2 should thus take center stage in pharmacological interventions.

## Introduction

With about 300 cases/100,000 people and mortality rates of 30–50% even in critical care units of developed countries^1^, sepsis is a life-threatening disease of high global abundance. Sepsis-associated liver dysfunction, often manifesting in form of cholestasis (i.e. a lack of bile secretion), is common in multiple organ dysfunctions and is associated with a poor prognosis^2^. Therefore, considerable efforts are currently made to define, to detect, to understand and maybe even to reverse especially the early stages of sepsis-associated liver dysfunction^3–5^.

Detoxification by bile formation and removal through canaliculae, bile ducts and the common hepatic duct, which then exits the liver and joins with the cystic and the pancreatic duct to enter the duodenum, is thought to depend on three major aspects: (i) an intact hepatocytic cytoskeleton that is essential for proper canalicular membrane surface modulations (microvilli decoration, contractions), (ii) proper cell junctions sealing off the canaliculae and (iii) proper functioning of hepatobiliary transport mechanisms, such as those mediated by the biliary transporter multidrug resistance-associated protein 2 (Mrp2)^5–7^. Disruptions of the above hepatocytic functions are thus considered as the major cellular hallmarks of cholestasis in sepsis^5,6^.

Hepatocytes are polarised cells. Their apical membranes form canaliculae for bile export. Their basolateral membrane is oriented towards the space of Disse (perisinusoidal space), which contains blood plasma from the fenestrated sinusoids. Both compartments are decorated by microvilli, which facilitate absorbance of plasma components from the fenestrated sinusoids and excretion into canaliculae, respectively.

Recently, it has been suggested that hepatocytic functions critically involved in cholestasis are affected by phosphatidylinositol 3-kinase (PI3K)-dependent signalling pathways, as PI3Kγ inhibition and knock-out (KO), respectively, protected from hepatic excretory dysfunction during early sepsis^8,9^.

PI3Kγ^10,11^ is highly expressed in white blood cells but also seems to occur in some other cells^12^ including hepatocytes^13^ at low levels. Yet, the cell biological processes regulated by PI3Kγ in hepatocytes remained largely unclear.

PI3Ks, once activated by various growth factors, hormones, and cytokines, phosphorylate phosphatidylinositol 4,5-bisphosphate (PIP_2_) at the 3-position and thereby generate phosphatidylinositol 3,4,5-trisphosphate (PIP_3_) at the plasma membrane^14^. A major component in PI3K signalling is Akt, which binds PIP_3_ through its pleckstrin homology (PH) domain. This leads to Akt activation via dual phosphorylation by the Phosphoinositide-dependent kinase 1 (PDK1) and the mechanistic target of rapamycin (mTOR)-rictor complex resulting in T308 and S473 phosphorylated Akt (pAkt)^15,16^. PI3K-mediated signalling based on the kinase activity of PI3Ks can be specifically addressed by replacing PI3Ks with kinase-dead (KD) mutants, while kinase-independent functions would not be affected by PI3K KD but only by PI3K KO^17,18^.

Apart from the thus far unknown functions of PI3Kγ in hepatocytes in general, it also remained to be demonstrated which of the cellular hallmarks of cholestasis in sepsis would be responsive to modulation of PI3K/Akt signalling. Therefore, we here use PI3K gain-of-function models as well as PI3Kγ KO and KD mice^17,18^ to study the role of PI3K signalling and PI3Kγ loss-of-function, respectively, in the cell biological defects underlying the three hallmarks of cholestasis in sepsis.

## Results

### PI3K signalling in cholestasis and demonstration of PI3Kγ expression in liver cells

**Figure 1:**
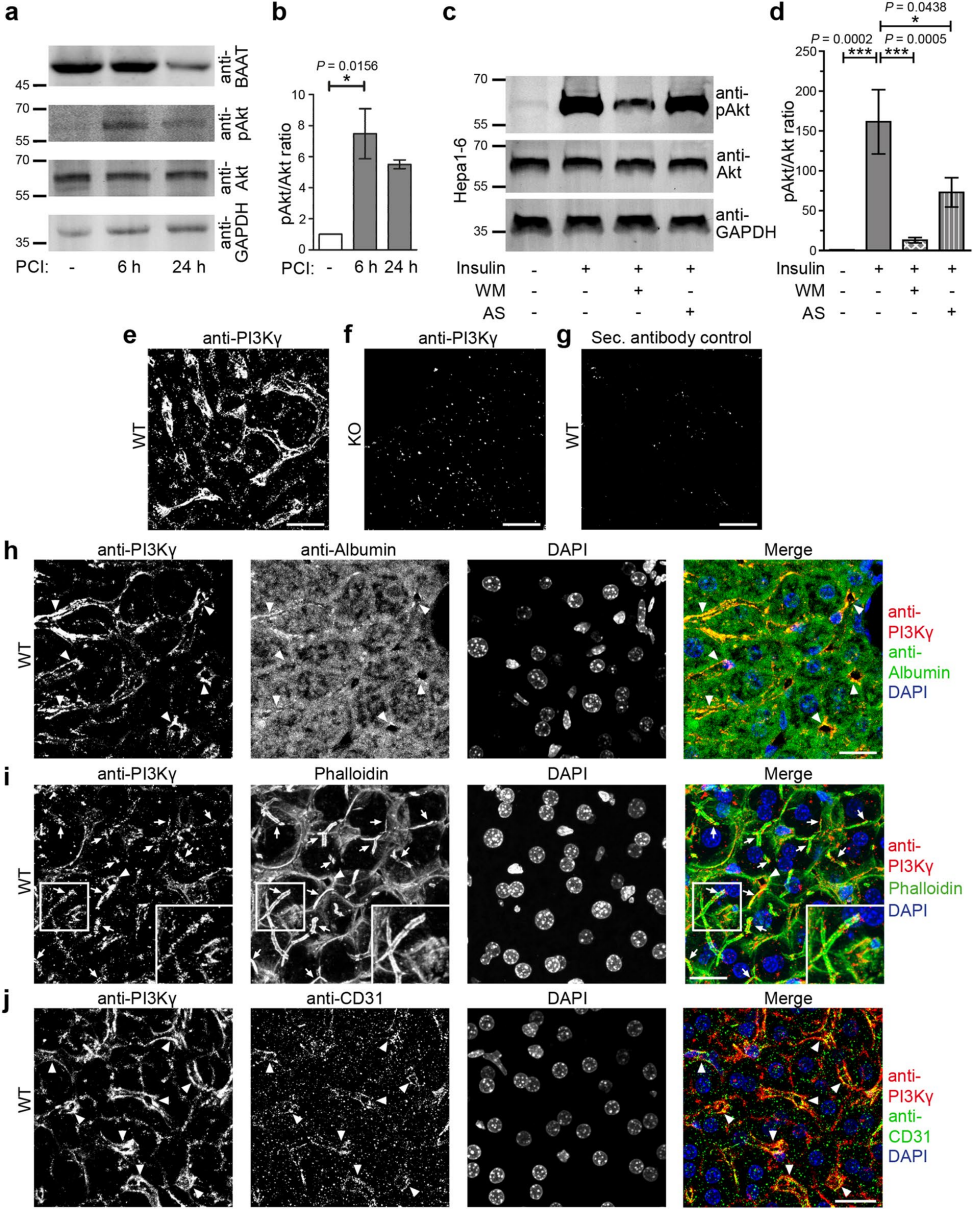
Visualisation of PI3Kγ in liver and detection of down-stream pAkt signalling. (**a**,**b**) Immunoblot analyses of liver extracts from control mice and mice subjected to sepsis by PCI (**a**) and quantitation of phospho-Akt (pAkt)/Akt levels normalised to sham control (**b**). (**c**,**d**) Anti-pAkt/Akt immunoblotting analyses (**c**) and quantitative analyses of Hepa1-6 cells stimulated with 100 nM insulin (5 min) and of Hepa1-6 cells stimulated with insulin after 1 h preincubation with the general PI3K inhibitor Wortmannin (WM, 100 nM) and the PI3Kγ inhibitor AS605240 (AS, 1 µM), respectively (**d**). Note that insulin-mediated pAkt signalling is completely dependent on PI3K and that a considerable portion of this signalling also is dependent on the PI3Kγ isoform in Hepa1-6 cells. (**e**–**g**) Anti-PI3Kγ immunofluorescence analysis of liver sections of wild-type (WT) (**e**) and PI3Kγ KO mice (**f**) and secondary antibody control (**g**) of a WT liver section. (**h**–**j**) Confocal images of anti-PI3Kγ (shown in red in merges) immunohistostainings of WT liver sections together with i) anti-albumin immunostaining (green in merge **h**) highlighting hepatocytes (**h**), with ii) phalloidin staining (shown in green in merge **i**) highlighting F-actin-rich structures (**i**), and with iii) an immunostaining for a marker for endothelial cells (CD31; green in merge **j**) (**j)**. Insets in (**i**) show enlargements of boxed areas in (**i**). DAPI in blue in merges. Arrows mark examples of F-actin and PI3Kγ-positive putative canalicular structures (1–2 µm in width). Arrowheads mark examples of perpendicularly and longitudinally cut large structures of about 6 µm in diameter (larger bile ducts or sinusoidal structures). Bars, 20 µm (**e**–**j**). Data, mean ± standard error of the mean (SEM). n = 4 each. Kruskal–Wallis + Dunn’s posttest (**b**); 1way ANOVA + Tukey’s posttest (**d**). *P < 0.05; ***P < 0.001.

Peritoneal contamination and infection (PCI) is a commonly used sepsis model that usually leads to multiple organ failure. Consistently, PCI samples we analysed showed declining levels of bile acid-CoA:amino acid *N*-acyltransferase (BAAT) (Fig. 1a) indicative of cholestasis^8^. PCI also led to activation of pAkt signalling in the liver (Fig. 1a,b), whereas apoptosis was not observed during the early phases of sepsis induction examined (Supplementary Fig. S1). The strong pAkt signalling supports the established view that PI3K-dependent signalling may play a role in processes that may eventually lead to hepatic dysfunctions. In line with this, PI3Kγ inhibition has been described as beneficial in cholestasis^8,9^. The hallmarks of cholestasis during sepsis are hepatocytic dysfunctions. This may imply that PI3Kγ has important functions in hepatocytes. Since whole liver homogenates and primary hepatocyte preparations will contain contaminations derived from e.g. immune and/or endothelial cells, we functionally tested for a role of PI3Kγ in a hepatocytic cell line. Insulin-triggered Akt activation in Hepa1-6 cells was completely suppressed by the PI3K inhibitor Wortmannin. Importantly, also the application of the PI3Kγ-selective inhibitor AS605240^19^ reduced PI3K/Akt signalling in Hepa1-6 cells. AS605240 led to pAkt/Akt ratios of insulin-stimulated cells that were not significantly different from control values of unstimulated cells anymore (Fig. 1c,d). Immunofluorescence analyses of mouse liver sections with anti-PI3Kγ antibodies including the use of PI3Kγ KO material as specificity controls also clearly demonstrated that PI3Kγ is expressed in liver tissue (Fig. 1e–g). Colocalisations with the hepatocyte marker albumin showed that PI3Kγ was present in hepatocytes (Fig. 1h).

PI3Kγ localisation patterns were mostly cortical and highlighted fine, often linear and sometimes branched structures. These PI3Kγ-positive structures were 1–2 µm in width and F-actin-rich, i.e. presumably represented canalicular segments decorated with microvilli (Fig. 1i; arrows). PI3Kγ furthermore occurred at circular structures of about 6 µm diameter (Fig. 1h-j; arrowheads), which also showed some F-actin enrichment (Fig. 1i) and represented larger bile ducts or sinusoidal structures.

Colabelling with CD31 as endothelial marker^20^ showed that PI3Kγ was not only expressed in hepatocytes but some part of the cortical PI3Kγ labelling reflected an additional expression in endothelial cells (Fig. 1j; arrowheads).

The observed PI3Kγ-specific signalling in Hepa1-6 cells and the PI3Kγ localisation to microvilli-decorated canaliculae suggested that PI3Kγ may indeed play a role in sepsis-relevant functions of hepatocytes.

### Stimulation of PI3K signalling has a moderate and short-lived effect on hepatocytic microvilli density in canaliculae

Three major hallmarks—(i) loss of microvilli in the canaliculae, (ii) disruption of canalicular tight junctions and (iii) impaired trans-hepatocytic transport—were suggested for sepsis and liver failure^5,6^, a lack of microvilli loss in wider canal structures that may have represented canaliculae was described upon PI3Kγ KO^8^ and we had observed PI3Kγ at cortical areas outlining F-actin-rich canaliculae (Fig. 1). We therefore first focused our detailed analyses on microvilli. We initially examined cultured cells under conditions that should lead to strong activation of class I PI3K/Akt signalling (Supplementary Fig. S2) as observed in PCI-induced sepsis (Fig. 1a,b). Both C5a and fMLP (*N*-formyl-l-methionyl-l-leucyl-l-phenylalanine) bind to G-protein-coupled receptors and lead to strong activation of class I PI3Ks in immune cells^21^. Also insulin reliably triggers PI3K class IA activity via the insulin receptor and insulin receptor substrates 1/2 (IRS1/2). In line with this, PI3Kγ is e.g. involved in the pathogenesis of obesity^22^. We also used lipopolysaccharides (LPS)—major bacterial membrane components^23^ that indirectly strongly activate Akt signalling^24^. However, none of these stimuli induced any obvious changes in dorsal membrane topology in Hepa1-6 cells or HepG2 cells. Quantitative determinations of the surface coverages by such protrusive membrane structures in control Hepa1-6 cells and in cells stimulated with insulin or with C5a or fMLP, which are known to activate PI3Kγ in immune cells^25,26^, confirmed that none of these PI3K/Akt signalling inducers caused any changes in microvilli-like membrane protrusions in Hepa1-6 cells (Supplementary Fig. S2d). This obviously could have different reasons, either PI3K/pAkt signalling was not successfully triggered in any of these conditions and/or microvilli formation and maintenance in cultured cells is not PI3K/pAkt-responsive at all and/or the cultured cells are not a suitable system for studying microvilli. pAkt/Akt analyses indeed showed that C5a or fMLP known to activate PI3Kγ in immune cells^25,26^ failed to induce pAkt activity (Supplementary Fig. S2e). Quantitative analyses confirmed that pAkt levels remained at base line (Supplementary Fig. S2f). However, insulin did result in a massive induction of PI3K/pAkt signalling (Supplementary Fig. S2e).

Furthermore, it became obvious that even using high resolution scanning electron microscopy (EM) it was impossible to clearly distinguish microvilli from filopodia, pseudovilli and other membrane protrusions in these cultured cells (Supplementary Fig. S2a–c). Apart from this, all cell lines showed a very heterogeneous membrane topology in both confluent and low-density cultures suggesting that none of these systems is suitable for detailed microvilli analyses.

**Figure 2:**
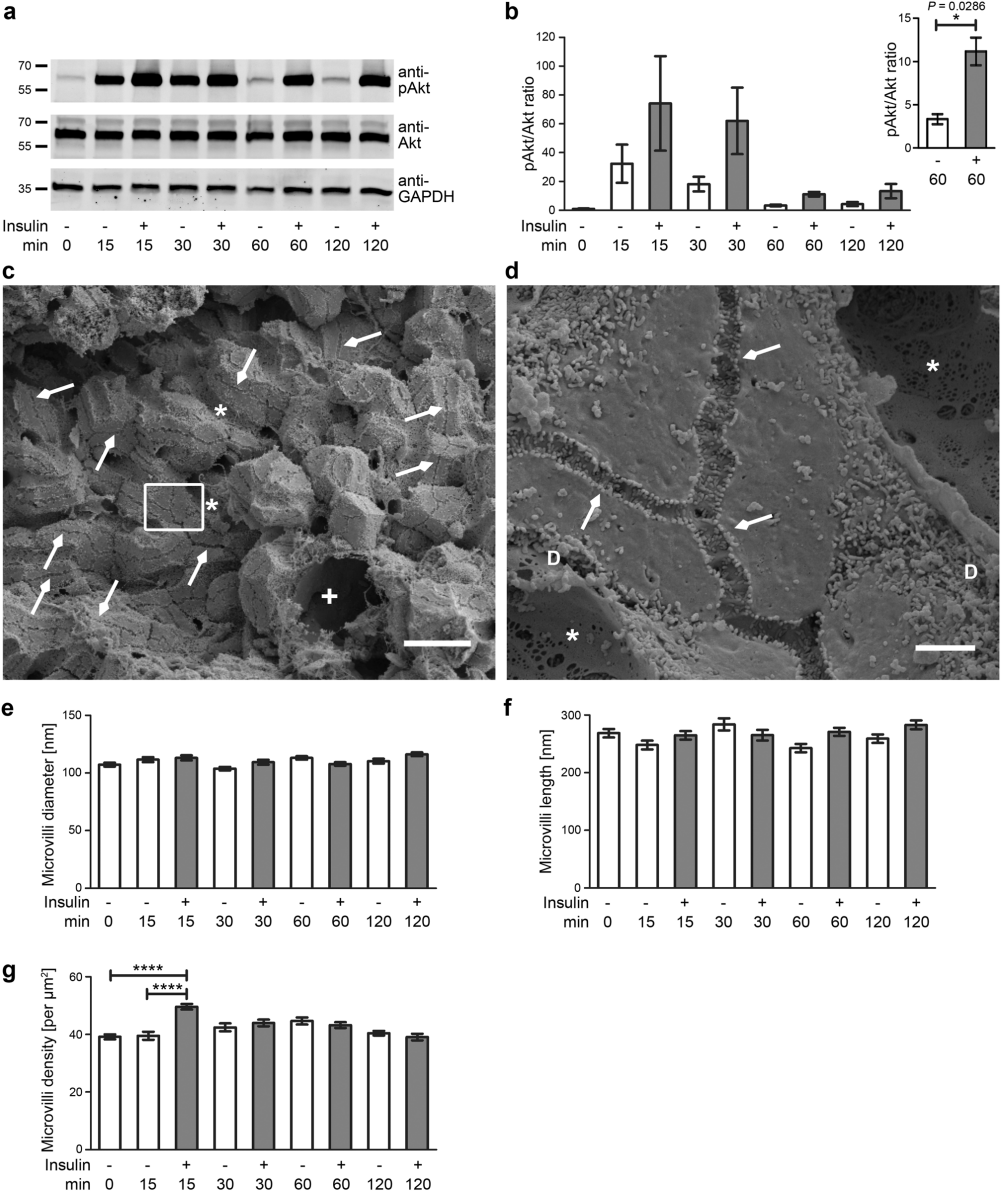
Microvilli of canaliculae in livers are unchanged upon induction of PI3K/Akt signalling. (**a**,**b**) Immunoblotting analyses reveal a sustained activation of pAkt signalling in perfused livers treated with 100 nM insulin (**a**). Quantitative analyses show the pAkt/Akt ratios over time normalised to control (0 min) (**b**). Note that also perfusions with buffer already led to a minor and transient activation of Akt signalling (presumably due to mechanostress) but that the insulin-induced effects were about 2–4 times as large and led to readily detectable, sustained pAkt immunosignals. Inset shows a comparison of pAkt/Akt ratios at 60 min using an adapted axis to visualise the prolonged pAkt signalling. n = 4 assays. (**c**,**d**) Scanning EM micrographs of WT liver tissue broken by shock-freezing in liquid nitrogen at low (**c**) and medium magnification (**d**; enlargement of boxed area in **c**). Note that low magnifications (**c**) provide an excellent overview over the liver tissue. Arrows mark examples of canaliculae broken open, asterisks mark examples of sinusoids (*) broken open and the cross marks a cross-break through a large blood vessel (+). At medium magnification (**d**), more delicate cellular structures, such as fenestrations in the sinusoids and microvilli, become visible and microvilli protruding into the Disse space (examples marked with “D”) can clearly be distinguished from canalicular microvilli (arrows), which were quantitatively evaluated. Bars, 20 µm (**c**); 2 µm (**d**). (**e**–**g**) Blinded, quantitative evaluations of canalicular microvilli diameter (**e**), length (**f**) and density (**g**) in WT mouse livers perfused with 100 nM insulin in KHB (grey columns) and KHB (Krebs Henseleit buffer) (control; white columns), respectively. n = 4 livers/condition à 12 pictures each; n = 48 canalicular regions of interest (ROIs) per condition (**g**) and n = 60 microvilli (**e**,**f**), respectively. Data, mean ± SEM. Unpaired t test and 2way ANOVA + Bonferroni’s test (**b**,**e**–**g**). *P < 0.05; ****P < 0.0001. For *P* < 0.0001, exact *P* values are not available. Other *P* values always are presented in the figures.

We thus next analysed real microvilli directly in the tissue. In order to achieve a strong activation of pAkt signalling, we established perfusions of livers with LPS and insulin, respectively. Apoptosis was not observed upon perfusion (Supplementary Fig. S3a). Control perfusions with buffer merely showed a minor, short-lasting effect of pAkt activation (Supplementary Fig. S3b,c; Fig. 2a), which may represent a transient Akt activation via mechanostress, as it was independent of buffers used (our unpublished data). In contrast, immunoblotting of homogenates of livers treated with LPS (100 ng/ml) and insulin (100 nM), respectively, showed a massive and long-lasting activation of pAkt signalling in the perfused livers (Supplementary Fig. S3b; Fig. 2a). Quantitative immunoblottings of liver homogenates confirmed that both the LPS and the insulin effect was strong (Supplementary Fig. S3c; Fig. 2b). Activations with insulin showed faster onsets and higher amplitudes of Akt signalling than LPS treatment. Already at 15 min, the pAkt/Akt values were 231% of the unstimulated control. At both 60 and 120 min, pAkt/Akt values were 325–367% of corresponding control values (Fig. 2b).

We therefore next analysed liver samples for the consequences of triggering such a high PI3K/Akt signalling on the hepatocytic hallmarks of cholestasis in sepsis. High resolution analyses of morphological structures in situ are often hampered by a lack of orientation in the tissue and by low comparability and require 3D information. We thus refrained from classical sectioning and transmission electron microscopy but used scanning EM on fixed liver samples broken by ultra-fast freezing (Fig. 2c,d). The obtained tissue fractures were large enough for the different purposes aimed for. First, it was possible to fully appreciate the cellular contexts within the liver. Second, it was very convenient to identify canaliculae, as breaks were often along canaliculae and usually not perpendicular to them (examples marked with arrows in Fig. 2c,d). Third, the method allowed for clearly distinguishing the delicate canalicular structures from larger bile ducts and from sinusoids with their discontinuous endothelium marked by fenestrations (examples marked with asterisks in Fig. 2c,d). Importantly, hepatocytic microvilli protruding into the space of Disse (examples marked with “D” in Fig. 2d) were also easily distinguishable from those protruding into canaliculae (Fig. 2d, arrows).

Halved canaliculae suitable for analyses were abundant in the liver samples (Fig. 2c). At medium magnifications they were traceable for long distances. This was important for quantitative microvilli density analyses, as more systematic analyses unveiled quite some heterogeneity in microvilli decoration along individual canaliculae (Fig. 2d).

A high number of canalicular microvilli was accessible for 3D assessments of individual microvillar properties when high magnifications were used. Although a massive increase of pAkt/Akt ratios was detected in livers perfused with insulin and pAkt levels remained more than 2–3 times above control for hours, no effects on microvilli morphologies were detected. Instead, as in untreated controls, their average diameter remained at about 110 nm and their average length remained at about 270 nm (Fig. 2e,f).

A very transient increase in microvilli density was observed upon 15 min of stimulation with insulin (+ 26%) but not for any other time point (Fig. 2g). The physiological relevance of this transient microvilli density increase remained unclear, as in sepsis the increased Akt signalling (Fig. 1a,b) was suggested to be accompanied with a loss of microvilli^5,6^. Furthermore, analyses of LPS-perfused livers at 15 min did not show any significant modulations of microvilli density (Supplementary Fig. S3d).

PI3K/Akt signalling induction thus did not lead to any loss of microvilli.

### Both PI3Kγ KO and ablation of its enzymatic activity have no detectable effects on liver tissue or on hepatocytic microvilli in canaliculae

We next evaluated whether microvilli decoration of canaliculae would in general be independent of PI3Kγ. Most class I PI3K functions depend on PIP_3_ generation. However, also kinase-independent functions exist^18,27^. We therefore analysed both PI3Kγ KO and KD mice^18^.

Examinations of hematoxylin and eosin (H&E)-stained liver tissues from different areas showed no obvious differences or structural defects when PI3Kγ KO and KD livers were compared to those of wildtype (WT) animals at light microscopic resolution. Further histopathology examinations, such as Elastica van Gieson (EvG), iron and periodic acid–Schiff (PAS) stainings, also did not show any obvious liver impairments (Supplementary Fig. S4).

**Figure 3:**
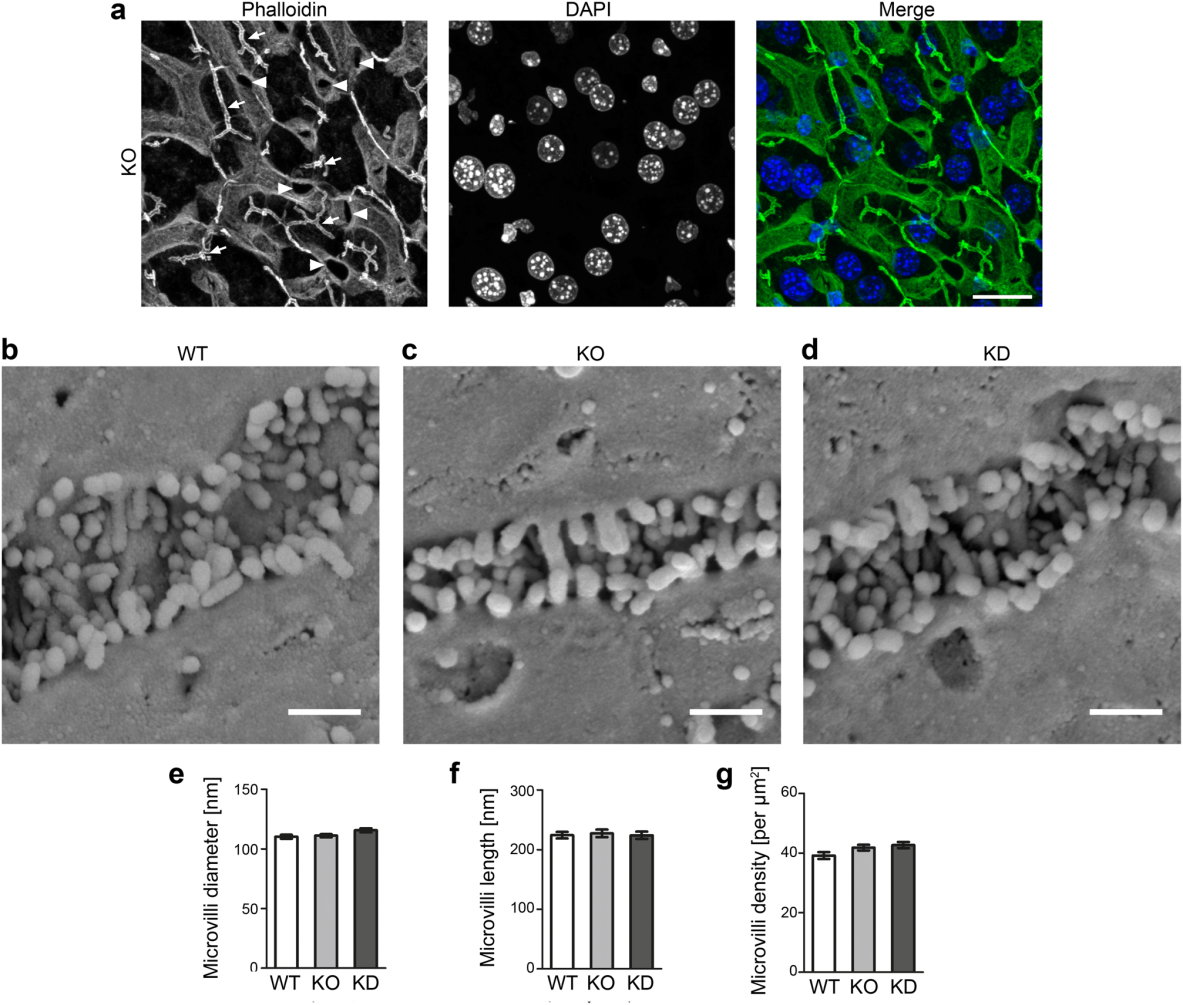
PI3Kγ KO does not result in any obvious organisational defects in liver tissue and also microvilli of canaliculae in livers are unchanged upon PI3Kγ loss-of-function. (**a**) Single fluorescent microscopy channels and a merged image of a liver section from a PI3Kγ KO mouse stained for F-actin with phalloidin (shown in green in merge) and with DAPI (blue in merge). Arrows mark examples of F-actin-positive putative canalicular structures (1–2 µm in width). Arrowheads mark examples of perpendicularly cut structures of about 6 µm diameter (larger bile ducts or sinusoidal structures). Bar, 20 µm. (**b**–**d**) Scanning EM images of livers from WT (**b**), PI3Kγ KO (**c**) and PI3Kγ KD mice (**d**). Bars, 500 nm. (**e**–**g**) Blinded, quantitative evaluations of microvilli diameter (**e**), length (**f**) and density in canalicular areas (**g**). n = 3 mice per genotype à 12 pictures, n = 36 canalicular ROIs (**g**) and n = 60 microvilli per genotype (**e**,**f**), respectively. Data, mean ± SEM. 1way ANOVA + Bonferroni’s test (**e**–**g**) (n.s.).

Confocal immunofluorescence analyses furthermore did not reveal any defects in F-actin-rich structures that mostly probably represent the different microvilli-decorated cell surfaces (Fig. 3a).

Also ultrastructural analyses of large segments of WT, PI3Kγ KO and PI3Kγ KD canaliculae using the same method established for the gain-of-function analyses (Fig. 2) did not reveal any obvious differences between microvilli-decorated canaliculae from mice of the three genotypes. Neither ablating the enzymatic activity of PI3Kγ (KD) nor complete PI3Kγ KO (additionally affecting PI3Kγ’s scaffolding function) led to any defects in the formation, organisation and/or maintenance of microvilli (Fig. 3b–d). Microvilli length, diameter and density all were indistinguishable when the three genotypes were compared in quantitative blinded analyses (Fig. 3e–g). Thus, PI3Kγ is not critical for proper formation, maintenance or structural organisation of canalicular microvilli.

Summarised from both experimental lines [gain-of-function (Fig. 2); loss-of-function (Fig. 3)], it can also be concluded that the beneficial effects observed in PI3Kγ KO mice during cholestasis induction^8^ seem not to relate to the first suggested major sepsis hallmark.

### Neither insulin-triggered, massive Akt signalling activation nor PI3Kγ KO and ablation of enzymatic activity by KD mutation have effects on tight junction integrity (second suggested sepsis hallmark)

We next evaluated the cellular function underlying the second sepsis hallmark, the disruption of canalicular tight junctions, for its responsiveness to PI3K/pAkt signalling and PI3Kγ, respectively.

**Figure 4:**
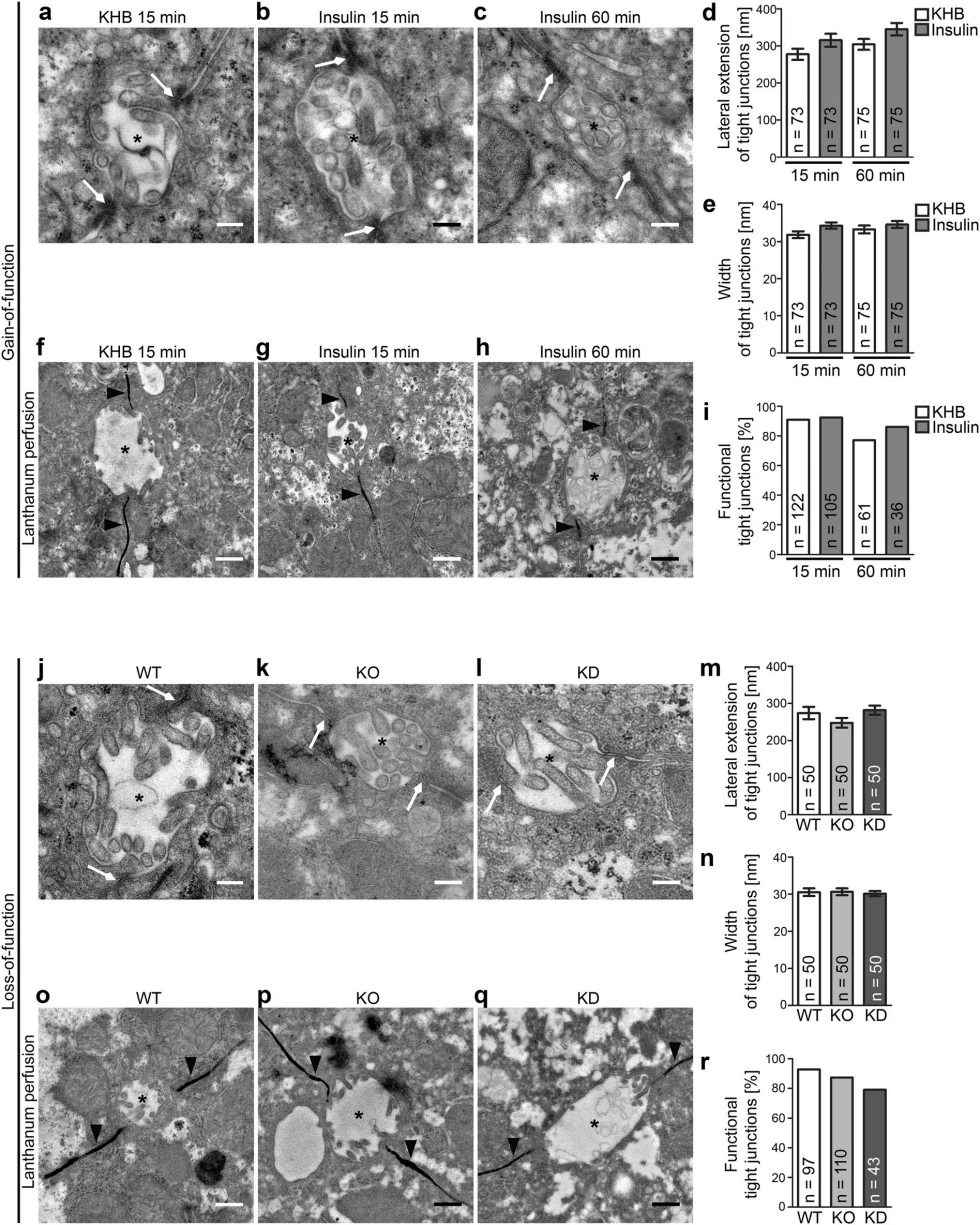
The second proposed sepsis hallmark, loss of canalicular integrity by disruption of tight junctions, also is independent of both PI3K gain-of-function and PI3Kγ loss-of-function. (**a**–**i**) PI3K gain-of-function analyses in liver sections of livers perfused by insulin. (**a**–**c**) Transmission EM images of araldite-embedded liver sections (60 nm) with canaliculae (*) and their tight junctions (arrows). Bars, 200 nm (**a**–**c**). (**d**,**e**) Blinded, quantitative evaluations of tight junctions [lateral extension, (**d**); width, i.e. close joint of the two plasma membranes, (**e**)]. (**f**–**i**) Analyses of tight junction functionality using lanthanum as electron-dense tracer filling sinusoids and spaces between hepatocytes membranes (arrowheads) but not reaching the lumen of canaliculae (*), provided that tight junctions are functional. Bars, 500 nm (**f**–**h**). (**i**) Blinded, quantitative analyses of tight junction functionality. (**j**–**l**) Electron micrographs of liver sections of WT (**j**), PI3Kγ KO (**k**) and PI3Kγ KD mice (**l**) with canaliculae (*) and their tight junctions (arrows). Bars, 200 nm (**j**–**l**). (**m**,**n**) Blinded, quantitative evaluations of individual tight junctions [lateral extension, (**m**); width, (**n**)]. (**o**–**q**) Analyses of tight junction functionality in WT (**o**), PI3Kγ KO (**p**) and PI3Kγ KD (**q**) liver using lanthanum as electron-dense tracer, which fills sinusoids and spaces between hepatocytes membranes (arrowheads) but does not reach the lumen of canaliculae (*) in case the tight junctions are functional. Bars, 500 nm (**o**–**q**). (**r**) Blinded, quantitative analyses of tight junction functionality. Data (**d**,**e**,**m**,**n**) mean ± SEM of individual tight junctions. Data (**i**,**r**) each percent of all canaliculae of all samples and animals. n numbers as indicated in (**d**,**e**,**i**,**m**,**n**,**r**). 2way ANOVA + Bonferroni’s test (**d**,**e**), n.s. 1way ANOVA + Bonferroni’s test (**m**,**n**), n.s. Blinded analyses.

Despite induced massive and prolonged PI3K/Akt signalling in perfused livers, transmission EM analyses of liver sections showed that tight junctions remained present and intact. The lateral extensions of the tight junctions were indistinguishable from the accompanying buffer controls at both 15 min and 60 min of stimulation and the membranes remained tightly aligned (total tight junction width, ~ 30 nm) (Fig. 4a–e).

We next used lanthanum perfusions^28^ to visualise whether tight junctions indeed remain functional and tight upon massive PI3K activation. Overview pictures clearly showed that lanthanum was observable in sinusoids and between hepatocytes membranes but that the electron-dense lanthanum did not reach the canaliculae but ended at the hepatocytic tight junctions (Fig. 4f–h). The finding that hepatocytic tight junctions remained fully functional was confirmed by quantitative analyses of stimulated livers compared to those merely perfused with Krebs Henseleit control buffer (KHB) (Fig. 4i).

Similar experiments were conducted for PI3K loss-of-function (Fig. 4j–r). Neither the scaffolding nor the enzymatic function of PI3Kγ was required for tight junction formation and/or maintenance in hepatocytes (Fig. 4j–l). Quantitative high-resolution analyses demonstrated that canalicular tight junctions had an undisturbed organisation in PI3Kγ KO and PI3Kγ KD livers, as their width and their lateral extension were unchanged (Fig. 4m,n).

Importantly, both PI3Kγ KO and PI3Kγ KD also clearly did not cause any defects in tight junction functionality, as administered lanthanum was effectively excluded from canaliculae (Fig. 4o–r).

Thus, our gain- and loss-of-function analyses showed that also the second proposed sepsis hallmark seems not to be directly linked to modulations of PI3K signalling in either direction.

### The third suggested sepsis hallmark, loss of canalicular transport activity as visualised by a loss of Mrp2 at the plasma membrane, is caused by stimulation of PI3K/Akt signalling

Detoxification by bile formation also relies on proper functioning of hepatobiliary transport mechanisms^5,6^. Absence of Mrp2 causes defects in the secretion of organic anions and congenital jaundice^7^. Sepsis but also phalloidin administration leads to sustained cholestasis in rats and is accompanied by a loss of Mrp2 from the canalicular membrane^29^. KO of the microvillar actin cytoskeletal component radixin has been suggested to result in reduced plasma membrane localisation of Mrp2^30^.

In our hands, immunolabellings of histological samples with the commercially available antibodies were not successful when compared to appropriate controls. As such analyses would anyway not provide enough resolution to clearly prove and quantify whether Mrp2 was integrated in the plasma membrane or would just be present in vesicles close to the plasma membrane, we instead set up a combination of freeze-fracturing and Mrp2 immunogold labelling—a method we recently established for quantitative ultrastructural examinations of plasma membrane-embedded proteins^31–35^. However, while we were successful with addressing such proteins in both brain^32^ and muscle material^34^, our attempts to obtain informative freeze-fractures of membranes from liver tissue and to additionally immunolabel such freeze-fracture replica with anti-Mrp2 antibodies failed.

**Figure 5:**
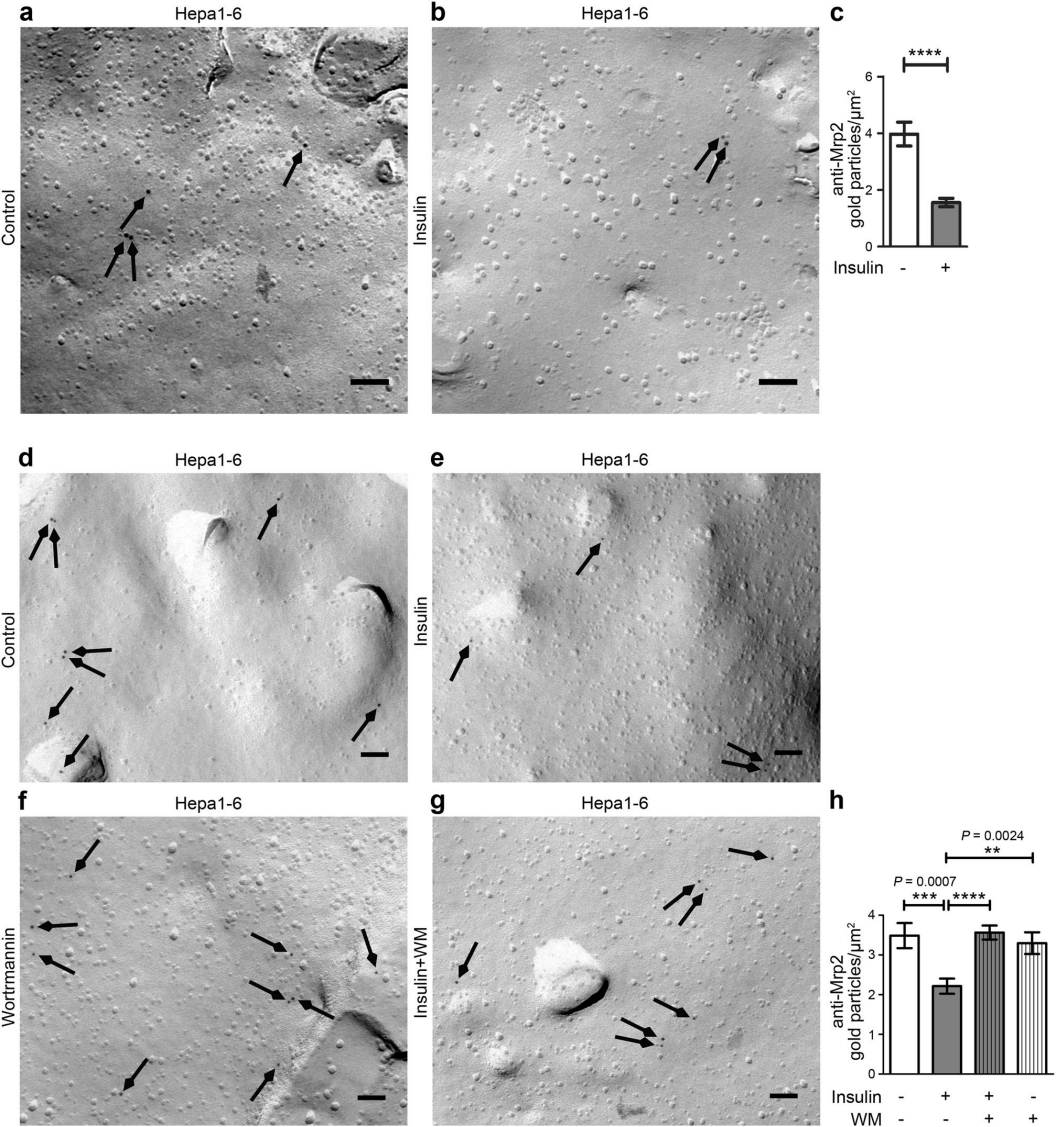
The third suggested sepsis hallmark, loss of canalicular transport activity, is brought about by strong PI3K/Akt signalling in hepatocytes. (**a**,**b**) Electron micrographs of freeze-fractured, anti-Mrp2-labelled unstimulated (control; **a**) and insulin-stimulated (**b**) Hepa1-6 cells. Bars, 100 nm. Arrows highlight examples of immunogold labelling. (**c**) Blinded, quantitative analyses of Mrp2 labelling densities at freeze-fractured plasma membranes of control and insulin-treated (100 nM; 5 min) Hepa1-6 cells. (**d**–**g**) Electron micrographs of freeze-fractured, anti-Mrp2-labelled unstimulated (control; **d**) and insulin-stimulated (**e**) as well as unstimulated and insulin-stimulated Hepa1-6 cells treated with the PI3K inhibitor Wortmannin (WM) (**f**,**g**). Bars, 100 nm. Arrows highlight examples of anti-Mrp2 immunogold labelling. (**h**) Blinded, quantitative analyses of Mrp2 labelling densities at freeze-fractured plasma membranes of the different conditions. Note that, as before (**a**,**b**), also the DMSO-containing (Wortmannin solvent) experimental set (**d**–**g**) shows the insulin-induced decline of anti-Mrp2 immunogold labelling density to about 2/µm^2^ and that this decline did not occur when Wortmannin was applied. All data, mean ± SEM. n = 58 (control) and 64 (insulin) images (**c**) and n = 54 (control), 50 (insulin), 49 (insulin + Wortmannin) and 29 (Wortmannin) images each (**h**) (3 µm^2^ analysed membrane area per image). Mann–Whitney (**c**) and Kruskal–Wallis + Dunn’s posttest (**h**). **P < 0.01; ***P < 0.001; ****P < 0.0001. For *P* < 0.0001, exact *P* values are not available. All other *P* values always are presented in the figures.

We thus next turned to Hepa1-6 cells for such analyses. We were able to freeze-fracture these cells effectively. Anti-Mrp2 immunolabelling was obtained specifically at fractured plasma membrane surfaces representing the membrane leaflet facing the cytoplasm (P-face). Strikingly, we observed that insulin treatment led to a significant reduction (*P* < 0.0001) of the density of anti-Mrp2 immunolabelling at the plasma membrane (Fig. 5a–c). More than 60% of the anti-Mrp2 labelling was lost upon stimulation of the cells (Fig. 5c; please also see Fig. 1c,d for PI3K/pAkt signalling being triggered in these samples).

The observed high PI3K/Akt signalling in insulin-treated Hepa1-6 (see Fig. 1c,d) does not prove that such a signalling is a reason for the drastic loss of Mrp2 from the plasma membrane induced by insulin. Instead, it could also be a mere coincidence. We therefore asked whether the loss of Mrp2 from the plasma membrane is PI3K signalling-dependent by using Wortmannin as inhibitor. Strikingly, the application of Wortmannin (in DMSO; controls correspondingly) completely suppressed the insulin-induced loss of Mrp2 from the plasma membrane (Fig. 5d–g). Quantitative analyses confirmed that the Mrp2 immunogold labelling density in Wortmannin-inhibited samples remained as high as in controls despite insulin stimulation, while insulin also in the new media conditions with DMSO still led to a highly significant (*P* = 0.0007 vs. control and *P* < 0.0001 vs. insulin + Wortmannin-treated cells) decline of Mrp2 levels at the plasma membrane (Fig. 5h).

Together, these results demonstrated that the third hallmark proposed for liver failure during sepsis, the impairment of trans-hepatocytic transport by the removal or loss of critical protein machinery from the plasma membrane, is a cellular process responsive to massive PI3K/Akt signalling.

### Loss of Mrp2 from the plasma membrane of hepatocytes upon Akt signalling stimulation is PI3Kγ-dependent

**Figure 6:**
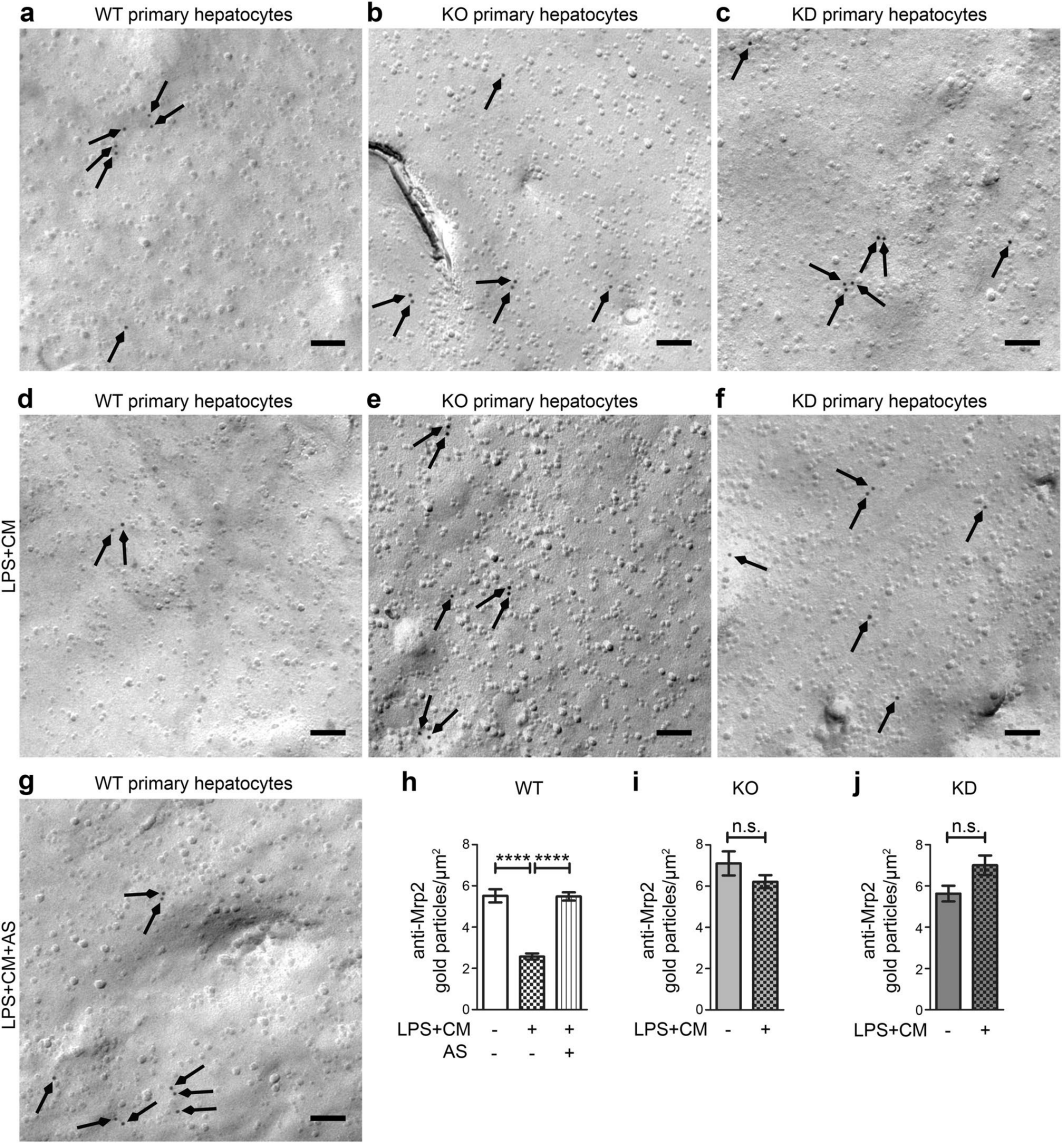
The PI3K/Akt signalling-induced loss of Mrp2 is dependent on PI3Kγ and its kinase activity and can thus be suppressed by either PI3Kγ loss-of-function or inhibition. (**a**–**g**) Electron micrographs of plasma membranes of freeze-fractured, anti-Mrp2-labelled primary hepatocytes from untreated WT (**a**), PI3Kγ KO (**b**) and KD (**c**) primary hepatocytes and examples from LPS and cytokine mix-treated (LPS + CM) primary hepatocytes isolated from mice of the three different genotypes (**d**–**f**) as well as an electron micrograph from LPS + CM and AS605240 (AS)-treated hepatocytes from WT mice (**g**). Arrows mark anti-Mrp2 immunogold labellings. Bars, 100 nm. (**h**–**j**) Quantitative evaluations of anti-Mrp2 immunogold labelling densities at membranes of the primary hepatocytes from WT, PI3Kγ KO and PI3Kγ KD mice comparing LPS + CM stimulated versus untreated hepatocytes [WT (**h**), KO (**i**), KD (**j**)] and versus LPS + CM and AS605240 (AS)-treated hepatocytes [WT (**h**)]. Data, mean ± SEM. n = 41–88 [(**h**) n = 70–88; (**i**) n = 57–70; (**j**) 41–71] pictures from three independent hepatocyte preparations/mice/each genotype and condition. Kruskal–Wallis + Dunn’s posttest (**h**), Mann–Whitney test (**i**,**j**; both n.s.). *P* < 0.0001. For *P* < 0.0001, exact *P* values are not available.

We next tested our results obtained in Hepa1-6 cell culture with primary hepatocytes isolated from mouse livers (Fig. 6a–j). We used an LPS-based stimulation mix [LPS + cytokine mix (CM)], as this may resemble even more closely the wealth of signalling pathways that are induced upon sepsis (for confirmation of pAkt signalling in the samples analysed, see Supplementary Fig. S5). Excitingly, the anti-Mrp2 labelling results we obtained in hepatocytes (Fig. 6) were in line with our previous observations in insulin-stimulated Hepa1-6 cells (Fig. 5). We again observed that Akt signalling stimulation led to a dramatic reduction of Mrp2 levels in the plasma membrane (Fig. 6a,d). Quantitative analyses demonstrated that the loss of Mrp2 from the plasma membrane indeed was dramatic (− 53%) and statistically highly significant (*P* < 0.0001) when compared to control (Fig. 6h).

We next tested whether the PI3K/Akt signalling-induced Mrp2 loss can be suppressed by specifically PI3Kγ KO. Strikingly, hepatocytes isolated from PI3Kγ KO mice were fully resistant to the PI3K/Akt signalling-induced loss of Mrp2. Irrespective of whether the PI3Kγ KO hepatocytes were stimulated or not, the Mrp2 immunogold labelling density remained at about 6/µm^2^, which also represented the WT levels (Fig. 6b,e,i).

Finally, we tested whether this effect, which may be highly beneficial in early sepsis, would be dependent on specifically the kinase function of PI3Kγ by using hepatocytes isolated from PI3Kγ KD mice. Also in PI3Kγ KD hepatocytes, Mrp2 levels at the plasma membrane were not suppressed by the induction of PI3K/Akt signalling but remained as high as in WT and control samples (Fig. 6c,f,j).

Since loss of the kinase activity was sufficient for suppressing the PI3K/Akt signalling-mediated Mrp2 loss from the plasma membrane, it seemed possible to preserve Mrp2 levels at the plasma membrane by pharmacological interventions using PI3Kγ-specific inhibitors. Application of AS605240 completely suppressed the Mrp2 loss from the plasma membrane of WT hepatocytes. The density of anti-Mrp2 immunolabelling remained at about 6/µm^2^, as in unstimulated hepatocytes from WT mice (Fig. 6g,h).

Our results clearly demonstrate that it is the third hepatocytic hallmark of cholestasis in sepsis, a loss of Mrp2 from the plasma membrane of hepatocytes, which is responsive to PI3K/Akt signalling. Since PI3Kγ KD conferred complete resistance to the loss of Mrp2 from the plasma membrane of hepatocytes and the same was observed for AS605240 application, it can furthermore be firmly concluded that it is the enzymatic function of PI3Kγ that is critical for this third suggested hepatocytic hallmark of cholestasis in sepsis.

## Discussion

Liver failure manifesting as cholestasis and jaundice is a critical component during sepsis-induced multiple organ failure. Even the short-term prognosis in these cases is very poor. Observations that PI3Kγ inhibition and KO, respectively, protects from hepatic excretory dysfunction during early sepsis^8,9^ suggested that, during the first hours when interventions may still be possible, hepatocytic functions critically involved in cholestasis are affected by PI3K/Akt-dependent signalling pathways. This may provide new therapeutic avenues, if it could be clarified which of the three cellular hallmarks of cholestasis in sepsis would be responsive to modulations of PI3K signalling. This required insights into the thus far largely uncharacterised functions of PI3Kγ in non-immune cells, such as hepatocytes. Surprisingly, using both gain- and loss-of-function paradigms in direct comparison our study demonstrated that only one of the three hepatocytic hallmarks of sepsis^6^ was PI3Kγ/Akt signalling-responsive.

Reports on putative effects of PI3K/Akt signalling induced by different means on tight junctions of different cellular systems are conflicting^36–41^. Our work shows that disruptions of canalicular tight junctions did neither occur upon massive PI3K/Akt signalling induction nor upon suppression of PI3Kγ signalling when this sepsis hallmark was quantitatively evaluated in a blinded study covering large and different parts of the livers. Even at ultra-high resolution, tight junctions were structurally unchanged. Furthermore they were fully functionally intact in PI3K/Akt gain-of-function and in PI3Kγ loss-of-function liver samples. Thus, this sepsis hallmark was not PI3K responsive and does therefore not represent one of the early phase disruptions that may be promising to be addressed therapeutically by modulations of PI3K signalling.

Given the fact that no systematic, quantitative and time-resolved data on tight junction integrity and functionality from large liver parts of septic patients is available, it is also well possible that the described loss of tight junction integrity and functionality merely represents a very late stage parameter of tissue dysfunctionality in an already failing liver.

Loss of canalicular microvilli is regarded as another important sepsis hallmark. Since PI3K signalling interfaces with the control of the actin cytoskeleton in multiple ways^42^, this sepsis hallmark was a strong candidate for being modulated by PI3K signalling. The second messenger PIP_3_ generated by PI3K activity^14^ recruits FYVE (Fab1, YOTB, Vac 1 and EEA1) zinc finger domain proteins and proteins containing PH domains with high affinity for PIP_3_ to the plasma membrane and promotes either their activation and/or their coclustering with other effector proteins^15,16,43^. Among those are guanine nucleotide exchange factors (GEFs) for Rho-type GTPases—major switches for controlling actin dynamics and organisation^44^.

It also seemed possible that conversion of apically enriched PIP_2_ to PIP_3_ by PI3K may partially disrupt cell polarity and thereby impair apical specialisations, such as microvilli. PIP_3_ was predominantly found in the inner leaflet of the basolateral membrane in epithelial cells^45^ and the PI3K substrate PIP_2_ plays an important role in priming and apical restriction of the actin-bundling protein ezrin^46,47^, which together with its relatives radixin and moesin was suggested to play a major role in microvilli formation and homeostasis^48^.

Yet, neither KO of PI3Kγ nor its replacement by a KD version caused any modulations of microvilli at the canalicular membrane of hepatocytes. Besides their density, also their length and diameter—morphological parameters, which in both microvilli and stereocilia strictly depend on F-actin dynamics and organisation^49,50^—were unchanged in both PI3Kγ KD and KO mice when compared to WT mice. Likewise, gain-of-function examinations did not reveal any changes in microvilli morphology. Only the density of canalicular microvilli showed a moderate and very short-lived response to insulin stimulations of livers. This observed moderate and very transient increase in microvilli density was not observed upon different stimulations of PI3K/Akt signalling in hepatocytic cells and is even opposite to the loss of microvilli expected as sepsis hallmark and also is opposite to the described reduction of microvilli in response to insulin in the small intestine of diabetic rats^51^. Therefore, changes in microvilli coverage of the canalicular membrane and organisation of microvilli clearly do not represent the beneficial effects brought about by PI3Kγ KO during sepsis.

In contrast, the third hallmark of sepsis, the rapid decline of Mrp2 levels from the plasma membrane of hepatocytes, massively responded to strong increases in PI3K/Akt signalling. Mrp2 is critical for proper bile flow^52^. The freeze-fracturing technique we applied specifically visualised plasma membrane-integrated Mrp2 and allowed for its quantitative determination. The levels of Mrp2 at the plasma membrane were reduced by 53–61% in both paradigms of PI3K/Akt signalling induction used (insulin and LPS supplemented with cytokines, respectively).

This PI3K/Akt signalling-induced effect is very well in line with experiments, in which cholestasis was induced by a variety of different paradigms^29,53–58^, although these studies either merely use biochemical fractionations and/or failed to provide any ultrastructural evidence of explicitly membrane-associated and canalicular Mrp2. Quantitative Western blotting of fractionations of livers of mice treated with phalloidin showed equal levels in the homogenates but a threefold increase of Mrp2 in microsome-enriched, i.e. endosomal compartment-containing, fractions^29^ and a 29% drop of Mrp2 levels in crude membrane preparations^59^, which presumably correspond to large fragments of the plasma membranes of the different liver cells. These alterations are thought to reflect a redistribution of Mrp2 by endocytic uptake^29,53,54,57^. Confocal microscopy showed that Mrp2 redistributed from areas outlined by the zonula occludens protein 1 (ZO-1) that may represent canalicular areas, to a broader distribution not so well confined by anti-ZO-1 immunoreactivity anymore when livers of septic mice and human patients were analysed^29,57,58^. Liver samples from mice treated with taurolithocholic acid to induce cholestasis also showed a reduction of Mrp2 immunosignals that were at least close to the plasma membrane^56^. Recent quantitation of overlaps of Mrp2 with F-actin at low resolution (20×) in tissue sections of mice subjected to PCI suggested that two thirds of the Mrp2 were leaving strongly F-actin-stained areas of the cell cortex that can be hypothesised to represent some microvilli-decorated cell surfaces^60^. Our immunolabellings of Mrp2 at freeze-fractured membranes and our quantitative analyses at ultra-high resolution analyses provided clear visual and quantitative evidence for changes of Mrp2 levels at the plasma membrane of both Hepa1-6 cells and hepatocytes in response to PI3K/Akt signalling and proved that these effects are explicitly dependent on the enzymatic activity of PI3Kγ.

Our finding that a loss of Mrp2 from the plasma membrane can be observed upon stimulation with LPS/cytokines but also with insulin may reflect one of the molecular/cell biological aspects associated with the dramatic failure of a clinical trial using an intensive insulin therapy of septic patients. Instead of having beneficial effects, the—at that time unexplainable—outcome was that insulin even increased the risk of organ failure and mortality^61^.

At the molecular level, changes of Mrp2 surface levels may involve ERM (ezrin, radixin, moesin) proteins. Radixin localises to canalicular microvilli and was shown to be part of Mrp2 protein complexes^30^. However, radixin also seems to accumulate at microvilli areas facing sinusoids^62^. Radixin KO mice are normal at birth but show a selective loss of Mrp2 from the canalicular membrane at the age of about 4 weeks. This slowly developing defect is accompanied by a hyperbilirubinemia and reminiscent of the Dubin–Johnson syndrome, which is caused by mutations in ABCC2 (human gene symbol for Mrp2)^30^.

It is also possible that crosstalk of PI3K pathways with Rho GTPase signalling pathways, which are thought to negatively control the phosphorylation-mediated F-actin binding of ERM proteins^63^, is underlying the observed PI3K-dependent modulation of Mrp2 distribution. Radixin’s constitutively dephosphorylated form (T564A) localised to canaliculae, whereas T564 phosphorylation of radixin did not only activate radixin’s F-actin binding but also directed it to the basolateral cortex of hepatocytes^64^. Such a crosstalk of PI3K pathways with Rho GTPase signalling pathways can easily be mediated by PIP_3_-responsive GEFs for Rho-type GTPases. This would be in line with the finding that it was sufficient to disrupt the enzymatic activity of PI3Kγ to maintain Mrp2 at the plasma membrane.

Taken together, our data demonstrates that the main therapeutic focus in reaping the observed benefits of PI3Kγ loss-of-function during early sepsis needs to be on the preservation of the availability of Mrp2 at the plasma membrane of hepatocytes. Our data argue that specifically inhibiting PI3Kγ in hepatocytes during early sepsis may represent an attractive strategy.

## Material and methods

### Mice

PI3Kγ KO and PI3Kγ KD mice lacking or expressing kinase-inactive PI3Kγ were bred on C57BL/6J background for more than 10 generations^17,18^. Control samples were taken from WT C57BL/6J mice. Livers were taken from 13 to 15 weeks old mice.

Induction of polymicrobial sepsis was performed by PCI, as described previously^65^. All experiments were performed in strict compliance with the EU guidelines for animal experiments and the committee of the Thuringian State Government on Animal Research approved the conducted animal experiments (TVA 02-015/13, TVA 02-007). The PCI liver homogenates evaluated in this study were biobank material derived from TVA 02-035/10.

Mice were housed under 14 h light/10 h dark conditions with ad libitum access to food and water in the central animal housing facility of the Jena University Hospital (*Zentrale Experimentelle Tierhaltung UKJ*).

### Antibodies

Primary antibodies used include mouse anti-Akt (1:1,000; Cell Signalling Technology), rabbit anti-phospho-Akt (1:1,000; Cell Signalling Technology), mouse anti-BAAT (ZA-18; 1:1,000; Santa Cruz Biotechnology), mouse anti-procaspase 9 (9508S; 1:1,000; Cell Signaling Technology), mouse anti-β-actin (A5316; 1:5,000, Sigma) and goat anti-GAPDH (1:1,000; Santa Cruz Biotechnology) for Western blot analysis as well as chicken anti-albumin (1:200; Abcam plc), rabbit anti-CD31 (1:50; Abcam plc) and mouse anti-PI3Kγ (1:1,000; Jena Bioscience GmbH) for immunohistochemistry. Rabbit anti-Mrp2 antibodies (1:50; Santa Cruz Biotechnology) were used for immunolabelling of freeze-fracture replica in combination with 10 nm gold-conjugated goat anti-rabbit secondary antibodies (1:50; British Biocell International).

Further secondary antibodies were horseradish peroxidase-labelled anti-rabbit and anti-mouse antibodies (0.1 μg/ml for Western blotting; from KPL). Additionally, DyLight800-conjugated goat anti-rabbit antibodies, Alexa Fluor680-labelled goat anti-mouse and donkey anti-goat antibodies, Alexa Fluor568-labelled donkey anti-mouse antibodies as well as Alexa Fluor488-labelled donkey anti-chicken, donkey anti-rabbit and donkey anti-mouse antibodies were used (1:10,000 for Western blotting and 1:1,000 for immunofluorescence analyses of tissue sections) (all purchased from Thermo Fisher Scientific Inc.).

### Cell culture and isolation of primary hepatocytes and generation of samples for Western blot and EM analyses

Primary hepatocyte isolations were done using a two-step collagenase perfusion procedure (modified from Refs.^66,67^). In brief, mice were anesthetised and killed by isoflurane overdose (5% volume). Liver perfusion was performed by cannulating the portal vein. After perfusion with Liver Perfusion Medium (Thermo Fisher Scientific Inc.) and subsequently with collagenase-containing Hepatocyte Liver Digest Medium (Thermo Fisher Scientific Inc.), the liver capsule lobes were mechanically disrupted in medium. The obtained suspension was purified with a 100 μm cell strainer. Williams’ E complete medium [10% (v/v) FCS, 1% (v/v) Pen/Strep, Merck Millipore] was added and the suspension was centrifuged. Subsequently, the pellet was washed with Williams’ E complete medium and Trypan blue staining was performed to determine the cell viability. Dead cells were removed by centrifugation with Percoll solution (Sigma-Aldrich). Viable hepatocytes were plated on collagen-coated plates (in Williams’ E complete medium), washed with phosphate-buffered saline (PBS) (2.68 mM KCl, 1.47 mM KH_2_PO_4_, 136.9 mM NaCl, 7.98 mM Na_2_HPO_4_, pH 7.5) after 3 h and then cultured in Williams’ E complete medium.

Hepa1-6 (CRL-1830, ATCC) and HepG2 cells (HB-8065, ATCC) were cultured in Dulbecco´s Modified Eagle Medium (DMEM, 10% (v/v) FCS, 1% Pen/Strep, Thermo Fisher Scientific Inc.).

For stimulation experiments, primary hepatocytes, Hepa1-6 and/or HepG2 cells were incubated in serum-depleted DMEM medium starting 2 h prior to stimulation. Insulin (100 nM, Sigma-Aldrich) was applied for 5 min.

Primary hepatocytes isolated from WT, PI3Kγ KO and PI3Kγ KD mice were furthermore stimulated with LPS (100 ng/ml; Sigma-Aldrich) supplemented with a cytokine mix containing TNF-α (50 ng/ml; ImmunoTools), IL-1β (10 ng/ml; ImmunoTools), IFN-γ (10 ng/ml; ImmunoTools) (LPS + CM) for quantitative determination of pAkt signalling by Western blotting and for analyses of Mrp2 plasma membrane levels using immunogold labelling of freeze-fracture replica of plasma membranes.

Inhibition experiments with primary hepatocytes and cell lines were done with the PI3K inhibitor Wortmannin (Selleck Chemicals) (100 nM, in 1% (v/v) DMSO final) and the PI3Kγ-specific inhibitor AS605240 (Selleck Chemicals) (1 µM and 1% (v/v) DMSO final for Hepa1-6; 500 nM and 0.5% (v/v) DMSO final for primary hepatocytes) 1 h prior to stimulation with insulin and LPS + CM, respectively.

Hepa1-6 cells were also stimulated for 5 min with 10 ng/ml C5a (ProSpec-Tany TechnoGene Ltd.), 1 µM fMLP (Sigma-Aldrich) or with 100 nM insulin in Dulbecco's modified Eagle's medium for quantitative determination of pAkt signalling by Western blotting and for blinded, quantitative evaluations of cell membrane areas covered by microvilli.

### Liver perfusion assays

Mice were sacrificed and the *inferior vena cava* was cannulated and long-term liver perfusion was performed with KHB (11.1 mM d-glucose, 0.9 mM MgSO_4_, 1.3 mM KH_2_PO_4_, 4.7 mM KCl, 118.2 mM NaCl, 2.5 mM CaCl_2_, 25.0 mM NaHCO_3_, pH 7.2) at a flow rate of approximately 8 ml/min applied for 15, 30, 60 and 120 min, respectively. Stimulations of Akt signalling pathways with insulin (100 nM) and LPS (100 ng/ml), respectively, were also done in KHB.

Subsequently, livers were either immediately homogenised for Western blot analyses or fixed for microscopical analyses.

### Histological examinations of liver tissues

Mouse livers were fixed with 4% (w/v) paraformaldehyde (PFA) and paraffin-embedded. Paraffin sections (4 mm) were prepared and subsequently stained according to standard procedures for H&E, EvG, PAS and iron staining^34^.

Liver sections were analysed by using a Zeiss Observer Z.1, a 20×/0.5 objective and AxioVision 4.8.2 software (Carl Zeiss AG).

### Western blot analysis

Mouse liver samples were homogenised with a Potter S Homogenisator (Sartorius AG) in ice-cold radioimmunoprecipitation assay (RIPA) buffer [50 mM Tris/HCl pH 8.0, 150 mM NaCl, 1% (v/v) IGEPAL CA-630 (Sigma-Aldrich), 0.5% (w/v) deoxycholate, 0.1% (w/v) SDS, 1 × Protease Inhibitor Cocktail complete and 1 × PhosphoStop (both from Roche Applied Science)].

Hepa1-6 cells and primary hepatocytes were collected from the plate and lysed in ice-cold RIPA buffer for 20–30 min.

Homogenates were centrifuged for 10 min at 10,000*g* at 4 °C. Supernatants of equal protein amounts (40 µg each) were separated by SDS-PAGE and blotted to polyvinylidenfluoride membranes.

Western blotting was performed using enhanced chemiluminescence detections. Quantitative Western blotting analyses were performed using a LI-COR Odyssey detection system (LI-COR Bioscience GmbH), as described before^68^. The ratio of pAkt/Akt levels was measured by Odyssey Infrared Imaging System Application Software Version 3.0.16 (LI-COR Bioscience GmbH).

### Immunohistochemistry

Mice were sacrificed by cervical dislocation. Livers were perfused with PBS and subsequently with 4% (w/v) PFA by cannulation of the *inferior vena cava*. The liver was then cut into pieces and the liver samples were fixed overnight in 4% (w/v) PFA at 4 °C. After washing with PBS, the liver samples were incubated overnight in 30% (w/v) sucrose, cut into cubes with 1 mm edge length and then frozen with 5% (w/v) sucrose.

Cryosections (14 µm) were generated using a Leica Cryostat CM3050 (Leica Biosystems Nussloch GmbH) and the sections blocked with 5% normal goat serum in phosphate buffer (PB; 77.4 mM Na_2_HPO_4_, 22.6 mM NaH_2_PO_4_, pH 7.4) containing 0.25% (v/v) Triton X-100 for 1 h. The sections were then incubated with primary antibodies in the above blocking buffer at 4 °C for 2 days. After washing with PB buffer the liver sections were incubated with secondary antibodies and/or phalloidin Alexa Fluor-568 (Thermo Fisher Scientific Inc.) (overnight at 4 °C). Subsequent to an additional DAPI staining, sections were embedded in Fluoromount-G (SouthernBiotech).

The fluorescently immunolabelled liver sections were analysed with a TCS SP5 confocal microscope (Leica) equipped with 63 × objectives and AxioVision software (Carl Zeiss AG).

### Sample preparation and scanning EM of liver samples and quantitative evaluations of microvilli

Liver perfusions were performed through the *inferior vena cava* using KHB and subsequently 4% (w/v) PFA. Pieces of mouse livers (3 mm × 3 mm× 3 mm) were fixed with 2.5% (v/v) glutaraldehyde and 4% (w/v) PFA in 0.1 M sodium cacodylate buffer pH 7.2 for 2 h. The samples were washed three times with 0.1 M sodium cacodylate buffer and then dehydrated with increasing ethanol concentrations (30, 50, 70, 80, 90 and 100%). At 70% (v/v) ethanol incubation, tissue breakage of the liver pieces was performed by quick-freezing the samples in liquid nitrogen.

Afterwards, all samples prepared for scanning EM were dried with a CPC 030 Critical Point Dryer (BAL-TEC) using liquid CO_2_ and coated with gold (approximately 2 nm coating thickness) by a SCD005 Sputter Coater (BAL-TEC).

Images were taken with a Zeiss Gemini scanning electron microscope (Carl Zeiss AG).

Blinded quantitative evaluations were performed by ImageJ (National Institutes of Health). In detail, canalicular areas that were fractured in halves in a longitudinal orientation were identified. Regions of interest (ROIs) of 2 µm in length and the width of the canaliculus were analysed for microvilli density expressed as microvilli per µm^2^ (apparent 2D) canalicular area.

Morphological parameters of individual microvilli (length and diameter) were also assessed in blinded quantitative evaluations. Longitudinal measurements were done at microvilli of exactly planar orientation.

As planar microvilli also offer a possibility to check whether the diameter is uniform over the entire length (which was the case for all microvilli analysed in detail), microvilli oriented in a planar manner were in fact also used for measurements of individual microvilli diameters, although in principle, diameters could have been determined from virtually every microvillus observed.

After analysis of the whole experiment, the samples were decoded and their data were averaged with samples of the same condition using Excel.

### Sample preparation and scanning EM of cultured cells

Cultured cells grown on coverslips were fixed with 2.5% (v/v) glutaraldehyde and 4% (w/v) PFA in 0.1 M sodium cacodylate buffer pH 7.2 for 1 h, washed three times with 0.1 M sodium cacodylate buffer, dehydrated and coated with gold as described above.

The surface of whole cells covered by microvilli was determined in a blinded manner by using the binarisation method, as described^69^.

### Ultrathin sectioning and transmission EM analysis

Livers were perfused, cut into pieces, fixed and washed as described for scanning EM (see above).

For the analyses of tight junction functionality, mouse livers were additionally perfused through the *inferior vena cava* with 3% (w/v) lanthanum nitrate in 4% (w/v) PFA for 10 min^28^ after short-term perfusion with 4% (w/v) PFA for 1 min.

The samples were prepared according to procedures described before^70^. In brief, for contrasting, liver samples were incubated with 1% (w/v) OsO_4_ for 2 h and washed three times with 0.1 M sodium cacodylate buffer. Samples were then dehydrated by rising ethanol concentrations and stained with 2% (w/v) uranylacetate in 50% (v/v) ethanol for 1 h before they were embedded in araldite resin at 60 °C for 48 h.

After ultrathin sectioning of the embedded samples using a LKB 8800A Ultratome III (LKB Produkter AB), the sections (60 nm) were placed on formvar-coated grids and were finally stained with 3% (w/v) lead citrate in ddH_2_O (Electron Microscopy Sciences) for 2 min.

The sections were investigated in an EM902A transmission electron microscope (Carl Zeiss AG) operated at 80 kV and images were recorded with a 1 k FastScan CCD camera (TVIPS camera and software).

### Immunogold labelling of freeze-fracture replica

Primary hepatocytes and Hepa1-6 cells were collected from the culture disk, centrifuged at 25 g for 3 min, quick-frozen, freeze-fractured, replicated and cleaned from attached cytosolic components according to procedures described previously^31,32,34^.

Freeze-fracture replica were then immunolabelled with rabbit anti-Mrp2 antibodies in PBS containing 1% (w/v) BSA, 0.5% (w/v) gelatine from cold water fish skin and 0.005% (v/v) Tween20 (overnight, 4 °C). After washing, samples were incubated with gold-conjugated goat anti-rabbit antibodies for 2 h at RT.

Immunolabelled, freeze-fractured plasma membranes were visualised by transmission EM using an EM902A and a 1 k FastScan CCD camera (see above).

Labelling densities were measured from 3 µm^2^ membrane ROIs representing one image each.

### Statistical analysis

Testing for normal data distribution and statistical analysis was done using Prism5 and Prism8 software (GraphPad Software).

Methods used for statistical significance calculations are stated in the figure legends. *P < 0.05, **P < 0.01, ***P < 0.001 and ****P < 0.0001 was used throughout.

## Supplementary information

Supplementary Information.

## Data Availability

All authors had access to all the data and have reviewed and approved the final manuscript.

## Abbreviations

BAAT: Bile acid-CoA:amino acid *N*-acyltransferase
EM: Electron microscopy
ERM: Ezrin, radixin, moesin
EvG: Elastica van Gieson
fMLP: *N*-Formyl-l-methionyl-l-leucyl-l-phenylalanine
GEF: Guanine exchange factor
H&E: Hematoxylin and eosin
IRS1/2: Insulin receptor substrates 1/2
KD: Kinase-dead
KHB: Krebs Henseleit buffer
KO: Knock-out
LPS: Lipopolysaccharides
Mrp2: Multidrug resistance-associated protein 2
mTOR: Mechanistic target of rapamycin
PAS staining: Periodic acid–Schiff staining
PBS: Phosphate buffered saline
PCI: Peritoneal contamination and infection
PDK1: Phosphoinositide-dependent kinase 1
PFA: Paraformaldehyde
PH domain: Pleckstrin homology domain
PIP_2_: Phospatidylinositol 4,5-bisphosphate
PIP_3_: Phosphatidylinositol 3,4,5-trisphosphate
PI3K: Phosphatidylinositol 3-kinase
RIPA buffer: Radioimmunoprecipitation assay buffer
ROI: Region of interest
SEM: Standard error of the mean
WM: Wortmannin
WT: Wildtyp

## References

[1] Martin GS, Mannino DM, Eaton S, Moss M (2003). The epidemiology of sepsis in the United States from 1979 through 2000. N. Engl. J. Med..

[2] Dellinger RP (2013). Surviving sepsis campaign: International guidelines for management of severe sepsis and septic shock: 2012. Crit. Care Med..

[3] Woznica EA, Inglot M, Woznica RK, Lysenko L (2018). Liver dysfunction in sepsis. Adv. Clin. Exp. Med..

[4] Nesseler N, Launey Y, Aninat C, Morel F, Mallédant Y, Seguin P (2012). Clinical review: The liver in sepsis. Crit. Care.

[5] Geier A, Fickert P, Trauner M (2006). Mechanisms of disease: Mechanisms and clinical implications of cholestasis in sepsis. Nat. Clin. Pract. Gastroenterol. Hepatol..

[6] Trauner M, Meier PJ, Boyer JL (1998). Molecular pathogenesis of cholestasis. N. Engl. J. Med..

[7] Paulusma CC (1996). Congenital jaundice in rats with a mutation in a multidrug resistance-associated protein gene. Science.

[8] Recknagel P (2012). Liver dysfunction and phosphatidylinositol-3-kinase signalling in early sepsis: Experimental studies in rodent models of peritonitis. PLoS Med..

[9] Martin EL (2010). Phosphoinositide-3 kinase γ activity contributes to sepsis and organ damage by altering neutrophil recruitment. Am. J. Respir. Crit. Care Med..

[10] Lopez-Ilasaca M, Crespo P, Pellici PG, Gutkind JS, Wetzker R (1997). Linkage of G protein-coupled receptors to the MAPK signaling pathway through PI 3-kinase γ. Science.

[11] Stoyanov B (1995). Cloning and characterization of a G protein-activated human phosphoinositide-3 kinase. Science.

[12] Jean S, Kiger AA (2014). Classes of phosphoinositide 3-kinases at a glance. J. Cell Sci..

[13] Hohenester S (2010). Phosphatidylinositol-3-kinase p110γ contributes to bile salt-induced apoptosis in primary rat hepatocytes and human hepatoma cells. J. Hepatol..

[14] Auger KR, Carpenter CL, Cantley LC, Varticovski L (1989). Phosphatidylinositol 3-kinase and its novel product, phosphatidylinositol 3-phosphate, are present in *Saccharomyces cerevisiae*. J. Biol. Chem..

[15] Chan TO, Rittenhouse SE, Tsichlis PN (1999). AKT/PKB and other D3 phosphoinositide-regulated kinases: Kinase activation by phosphoinositide-dependent phosphorylation. Annu. Rev. Biochem..

[16] Toker A, Cantley LC (1997). Signalling through the lipid products of phosphoinositide-3-OH kinase. Nature.

[17] Hirsch E (2000). Central role for G protein-coupled phosphoinositide 3-kinase γ in inflammation. Science.

[18] Patrucco E (2004). PI3Kγ modulates the cardiac response to chronic pressure overload by distinct kinase-dependent and -independent effects. Cell.

[19] Camps M (2005). Blockade of PI3Kγ suppresses joint inflammation and damage in mouse models of rheumatoid arthritis. Nat. Med..

[20] Albelda SM, Muller WA, Buck CA, Newman PJ (1991). Molecular and cellular properties of PECAM-1 (endoCAM/CD31): A novel vascular cell–cell adhesion molecule. J. Cell. Biol..

[21] Hawkins PT, Stephens LR, Suire S, Wilson M (2010). PI3K signaling in neutrophils. Curr. Top. Microbiol. Immunol..

[22] Beretta M, Bauer M, Hirsch E (2015). PI3K signaling in the pathogenesis of obesity: The cause and the cure. Adv. Biol. Regul..

[23] Raetz CR, Whitfield C (2002). Lipopolysaccharide endotoxins. Annu. Rev. Biochem..

[24] Aderem A, Ulevitch RJ (2000). Toll-like receptors in the induction of the innate immune response. Nature.

[25] Stephens L, Jackson T, Hawkins PT (1993). Synthesis of phosphatidylinositol 3,4,5-trisphosphate in permeabilized neutrophils regulated by receptors and G-proteins. J. Biol. Chem..

[26] Coffer PJ, Schweizer RC, Dubois GR, Maikoe T, Lammers JW, Koenderman L (1998). Analysis of signal transduction pathways in human eosinophils activated by chemoattractants and the T-helper 2-derived cytokines interleukin-4 and interleukin-5. Blood.

[27] Rauch J, Volinsky N, Romano D, Kolch W (2011). The secret life of kinases: Functions beyond catalysis. Cell Commun. Signal.

[28] Goodenough DA, Revel JP (1970). A fine structural analysis of intercellular junctions in the mouse liver. J. Cell. Biol..

[29] Rost D, Kartenbeck J, Keppler D (1999). Changes in the localization of the rat canalicular conjugate export pump Mrp2 in phalloidin-induced cholestasis. Hepatology.

[30] Kikuchi S (2002). Radixin deficiency causes conjugated hyperbilirubinemia with loss of Mrp2 from bile canalicular membranes. Nat. Genet..

[31] Koch D, Westermann M, Kessels MM, Qualmann B (2012). Ultrastructural freeze-fracture immunolabeling identifies plasma membrane-localized syndapin II as a crucial factor in shaping caveolae. Histochem. Cell Biol..

[32] Schneider K (2014). ProSAP1 and membrane nanodomain-associated syndapin I promote postsynapse formation and function. J. Cell Biol..

[33] Zobel T (2015). Cooperative functions of the two F-BAR proteins Cip4 and Nostrin in the regulation of E-cadherin in epithelial morphogenesis. J. Cell Sci..

[34] Seemann E (2017). Deciphering caveolar functions by syndapin III KO-mediated impairment of caveolar invagination. Elife.

[35] Wolf D (2019). Ankyrin repeat-containing N-Ank proteins shape cellular membranes. Nat. Cell Biol..

[36] Gonzalez-Mariscal L, Tapia R, Chamorro D (2008). Crosstalk of tight junction components with signaling pathways. Biochim. Biophys. Acta.

[37] Khan N, Binder L, Pantakani DVK, Asif AR (2017). MPA modulates tight junctions' permeability via midkine/PI3K pathway in Caco-2 cells: A possible mechanism of leak-flux diarrhea in organ transplanted patients. Front. Physiol..

[38] Sheth P, Basuroy S, Li C, Naren AP, Rao RK (2003). Role of phosphatidylinositol 3-kinase in oxidative stress-induced disruption of tight junctions. J. Biol. Chem..

[39] Lin N, Xu LF, Sun M (2013). The protective effect of trefoil factor 3 on the intestinal tight junction barrier is mediated by toll-like receptor 2 via a PI3K/Akt dependent mechanism. Biochem. Biophys. Res. Commun..

[40] Yu C (2016). The effects of glucagon-like peptide-2 on the tight junction and barrier function in IPEC-J2 cells through phosphatidylinositol 3-kinase-protein kinase B-mammalian target of rapamycin signaling pathway. Asian Aust. J. Anim. Sci..

[41] Shao Y, Wolf PG, Guo S, Guo Y, Gaskins HR, Zhang B (2017). Zinc enhances intestinal epithelial barrier function through the PI3K/AKT/mTOR signaling pathway in Caco-2 cells. J. Nutr. Biochem..

[42] Lien EC, Dibble CC, Toker A (2017). PI3K signaling in cancer: Beyond AKT. Curr. Opin. Cell Biol..

[43] Warfel NA, Niederst M, Newton AC (2011). Disruption of the interface between the pleckstrin homology (PH) and kinase domains of Akt protein is sufficient for hydrophobic motif site phosphorylation in the absence of mTORC2. J. Biol. Chem..

[44] Qian Y (2004). PI3K induced actin filament remodeling through Akt and p70S6K1: Implication of essential role in cell migration. Am. J. Physiol. Cell Physiol..

[45] van den Bogaart G (2011). Membrane protein sequestering by ionic protein–lipid interactions. Nature.

[46] Fievet BT (2004). Phosphoinositide binding and phosphorylation act sequentially in the activation mechanism of ezrin. J. Cell Biol..

[47] Pelaseyed T, Viswanatha R, Sauvanet C, Filter JJ, Goldberg ML, Bretscher A (2017). Ezrin activation by LOK phosphorylation involves a PIP_2_-dependent wedge mechanism. Elife.

[48] Saotome I, Curto M, McClatchey AI (2004). Ezrin is essential for epithelial organization and villus morphogenesis in the developing intestine. Dev. Cell.

[49] Mooseker MS (1985). Organization, chemistry, and assembly of the cytoskeletal apparatus of the intestinal brush border. Annu. Rev. Cell Biol..

[50] Rzadzinska AK, Schneider ME, Davies C, Riordan GP, Kachar B (2004). An actin molecular treadmill and myosins maintain stereocilia functional architecture and self-renewal. J. Cell Biol..

[51] Williams M, Mayhew TM (1992). Responses of enterocyte microvilli in experimental diabetes to insulin and an aldose reductase inhibitor (ponalrestat). Virchows Arch. B Cell. Pathol. Incl. Mol. Pathol..

[52] Smit JJ (1993). Homozygous disruption of the murine mdr2 P-glycoprotein gene leads to a complete absence of phospholipid from bile and to liver disease. Cell.

[53] Trauner M (1997). The rat canalicular conjugate export pump (Mrp2) is down-regulated in intrahepatic and obstructive cholestasis. Gastroenterology.

[54] Kubitz R, Wettstein M, Warskulat U, Haussinger D (1999). Regulation of the multidrug resistance protein 2 in the rat liver by lipopolysaccharide and dexamethasone. Gastroenterology.

[55] Paulusma CC (2000). Zonal down-regulation and redistribution of the multidrug resistance protein 2 during bile duct ligation in rat liver. Hepatology.

[56] Beuers U (2001). Tauroursodeoxycholic acid inserts the apical conjugate export pump, Mrp2, into canalicular membranes and stimulates organic anion secretion by protein kinase C-dependent mechanisms in cholestatic rat liver. Hepatology.

[57] Mottino AD, Cao J, Veggi LM, Crocenzi F, Roma MG, Vore M (2002). Altered localization and activity of canalicular Mrp2 in estradiol-17β-d-glucuronide-induced cholestasis. Hepatology.

[58] Kojima H, Sakurai S, Yoshiji H, Uemura M, Yoshikawa M, Fukui H (2008). The role of radixin in altered localization of canalicular conjugate export pump Mrp2 in cholestatic rat liver. Hepatol. Res..

[59] Saeki J, Sekine S, Horie T (2011). LPS-induced dissociation of multidrug resistance-associated protein 2 (Mrp2) and radixin is associated with Mrp2 selective internalization in rats. Biochem. Pharmacol..

[60] Schaarschmidt B (2018). Molecular signatures of liver dysfunction are distinct in fungal and bacterial infections in mice. Theranostics.

[61] Brunkhorst FM (2008). Intensive insulin therapy and pentastarch resuscitation in severe sepsis. N. Engl. J. Med..

[62] Amieva MR, Wilgenbus KK, Furthmayr H (1994). Radixin is a component of hepatocyte microvilli in situ. Exp. Cell Res..

[63] Matsui T (1998). Rho-kinase phosphorylates COOH-terminal threonines of ezrin/radixin/moesin (ERM) proteins and regulates their head-to-tail association. J. Cell Biol..

[64] Suda J, Zhu L, Karvar S (2011). Phosphorylation of radixin regulates cell polarity and Mrp-2 distribution in hepatocytes. Am. J. Physiol. Cell Physiol..

[65] Gonnert FA (2012). Hepatic Fibrosis in a long-term murine model of sepsis. Shock.

[66] Goncalves LA, Vigario AM, Penha-Goncalves C (2007). Improved isolation of murine hepatocytes for in vitro malaria liver stage studies. Malar. J..

[67] Braccini L (2015). PI3K-C2γ is a Rab5 effector selectively controlling endosomal Akt2 activation downstream of insulin signalling. Nat. Commun..

[68] Schwintzer L, Koch N, Ahuja R, Grimm J, Kessels MM, Qualmann B (2011). The functions of the actin nucleator Cobl in cellular morphogenesis critically depend on syndapin I. EMBO J..

[69] Julio G, Merindano MD, Canals M, Ralló M (2008). Image processing techniques to quantify microprojections on outer corneal epithelial cells. J. Anat..

[70] Beer AJ, González Delgado J, Steiniger F, Qualmann B, Kessels MM (2020). The actin nucleator Cobl organises the terminal web of enterocytes. Sci. Rep..

