# Supplementary material for: Reduced Mrp2 surface availability as PI3Kγ-mediated hepatocytic dysfunction reflecting a hallmark of cholestasis in sepsis
Source: Sci Rep. 2020 Aug 4;10:13110. doi: 10.1038/s41598-020-69901-3 (PMC7403153; doi:10.1038/s41598-020-69901-3)
Supplement: Supplementary file 1 — Supplementary Information. [file 41598_2020_69901_MOESM1_ESM.pdf]

Supplementary Information

Reduced Mrp2 surface availability

as PI3Kγ-mediated hepatocytic dysfunction reflecting a hallmark of cholestasis in sepsis

Anne J. Beer[1], David Hertz[1,5,7], Eric Seemann[1,7], Martina Beretta[2,3,6], Martin Westermann[4], Reinhard Bauer[3], Michael Bauer[2], Michael M. Kessels[1]*, Britta Qualmann[1]*

[1] Institute of Biochemistry I,

[2] Center for Sepsis Control and Care & Department of Anesthesiology and Intensive Care Medicine,

[3] Institute of Molecular Cell Biology, CMB-Center for Molecular Biomedicine, and

[4] Electron Microscopy Center,

Jena University Hospital – Friedrich Schiller University Jena, 07743 Jena, Germany

[5] present address: Research Center Borstel, Leibniz Lung Center, Priority Area Infections, Parkallee 1-40, 23845 Borstel, Germany

[6] present address: School of Biotechnology and Biomolecular Sciences, University of New South Wales Sydney, Australia

[7] contributed equally

* Correspondence: Britta.Qualmann@med.uni-jena.de & Michael.Kessels@med.uni-jena.de

Supplementary Figures and Legends of Supplementary Figures

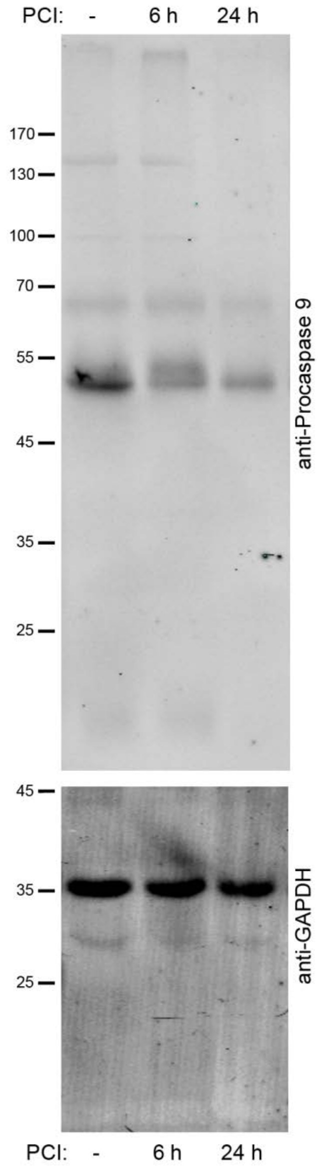

Figure S1. No signs of apoptosis in murine liver homogenates during early phases of sepsis induced by PCI.

Immunoblot analyses of liver extracts from sham mice (-) and mice subjected to sepsis by PCI after 6 h and 24 h. Upper panel, anti-procaspase 9 immunoblotting to screen for putative apoptosis. Note that procaspase 9 (band at about 50 kDa) was successfully detected but cleavage products (activated caspase 9; about 37 kDa) indicative of apoptosis induction were absent. Anti-GAPDH immunoblotting served as loading control.

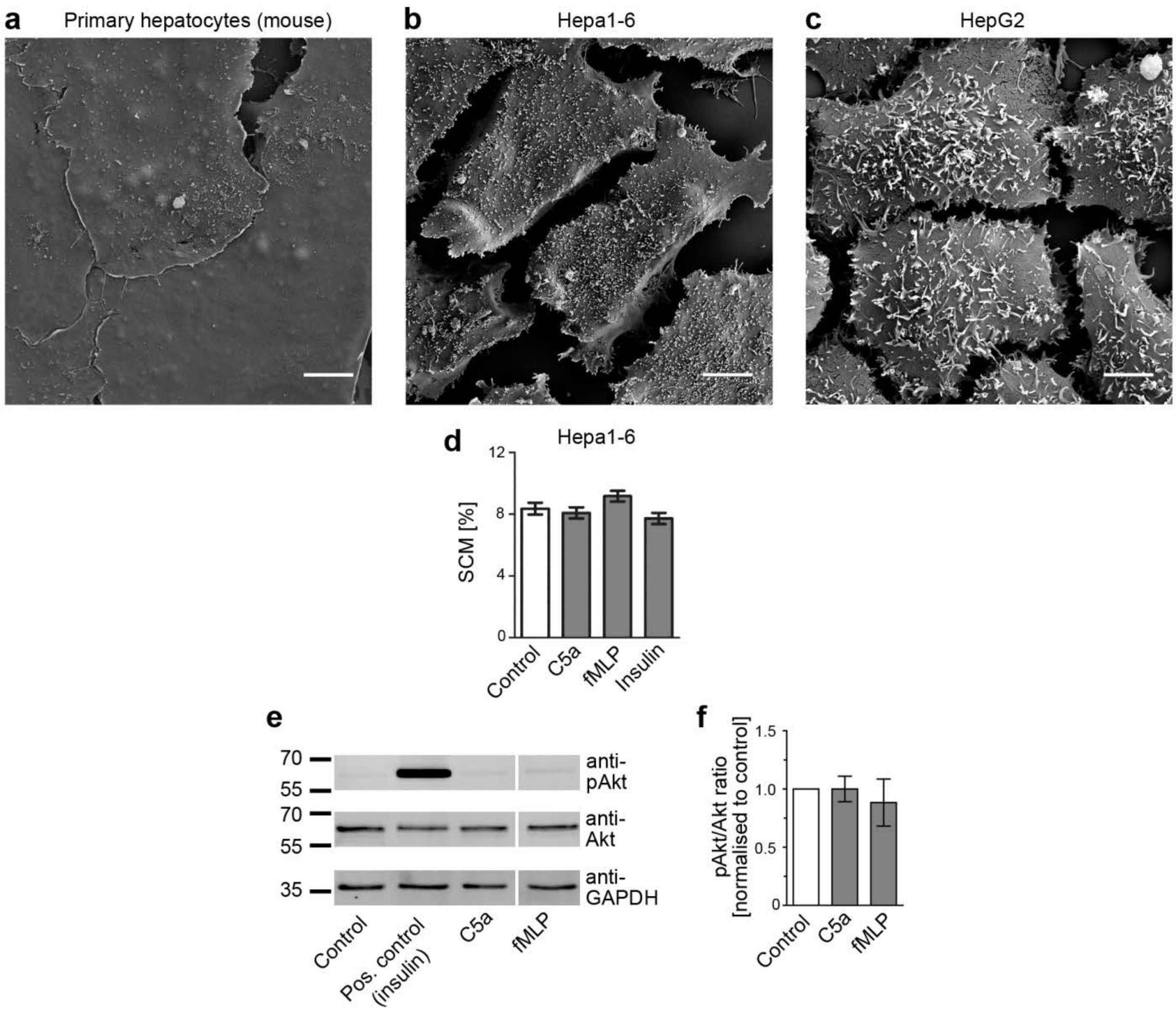

Figure S2. PI3Kγ activity modulations do not lead to changes in cell membrane topology of Hepa1-6 cells.

a-c, Scanning EM of primary hepatocytes (**a**), Hepa1-6 cells (**b**) and HepG2 cells (**c**). Bars, 5 µm. **d,** Blinded, quantitative evaluations of the cell surface of Hepa1-6 cells covered by microvilli (SCM) in percent of total area. Stimulations (5 min) with 10 ng/ml C5a, 1 µM fMLP, 100 nM insulin versus control. n=60 cells per condition from 3 independent cellular assays. Data, mean±SEM. 1way ANOVA + Bonferroni's test; n.s. **e,f,** Anti-pAkt/Akt immunoblotting analyses (**e**) of Hepa1-6 cells stimulated for 5 min with 100 nM insulin (positive control) as well as with C5a and fMLP, respectively, and quantitative analyses thereof (**f**). Note that neither C5a nor fMLP, two reagents known to activate specifically PI3Kγ in immune cells, was able to trigger pAkt signalling in Hepa1-6 cells, as unveiled by anti-pAkt Western blotting (**e**; upper panel; white line

represents lanes of the blot that were omitted) as well as by high-sensitivity comparison to the also very low pAkt levels in control cells (**f**). n=5 independent experiments. Data, mean±SEM.

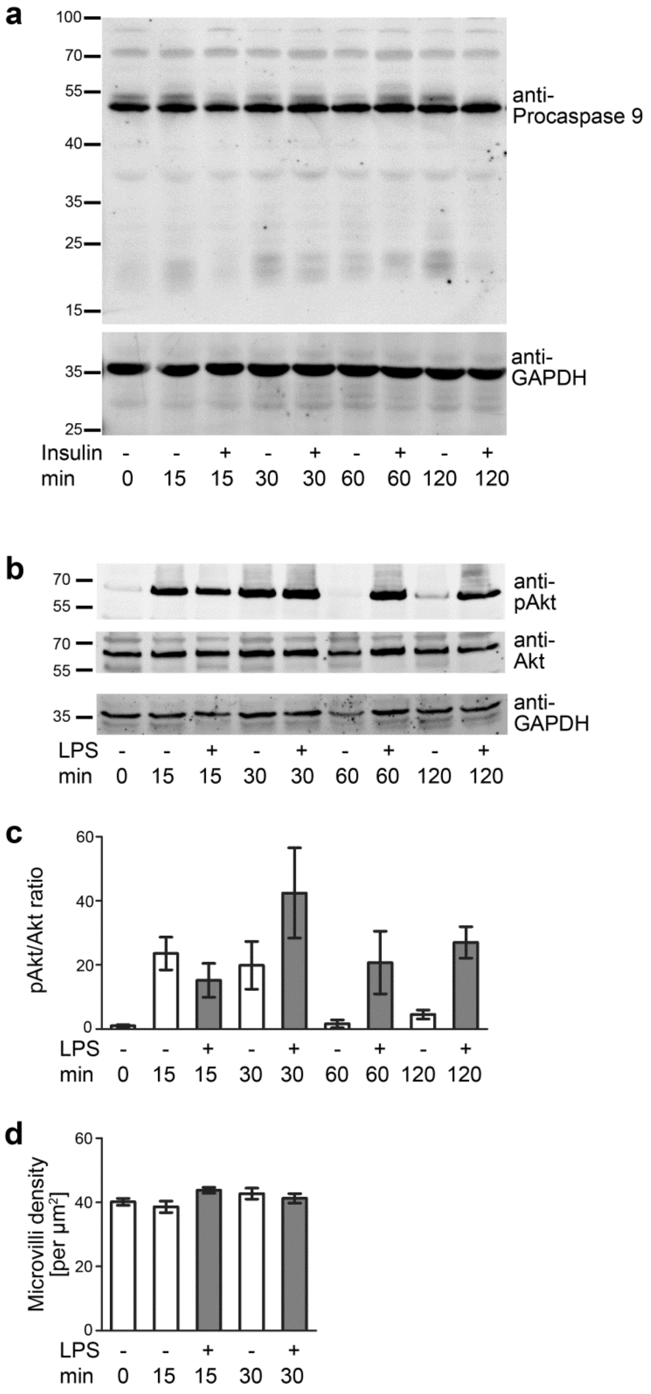

Figure S3. Activation of Akt signalling in livers stimulated with LPS.

a, Anti-procaspase 9 immunoblotting analyses of homogenates of livers perfused with insulin (100 nM) showing solely the procaspase at about 50 kDa but no proteolytic cleavage products (caspase 9) of about 37 kDa and below, which would have been indicative of apoptosis induction. **b,** Immunoblotting analyses of homogenates of livers perfused with 100 ng/ml LPS showed late and weaker activation of pAkt signalling compared to insulin perfusion (**Figure 2**). **c,** Quantitative

analyses show the pAkt/Akt ratios over time normalised to control (0 min). n=3 assays (**c**). **d,** Blinded, quantitative evaluation of canalicular microvilli density from scanning EM images of WT mouse livers perfused with 100 ng/ml LPS and KHB control, respectively, for 15 min (the highest pAkt/Akt value during insulin stimulation of PI3K/Akt signalling (**Figure 2a,b**)) and for 30 min (corresponding to the highest pAkt/Akt value for LPS stimulation of PI3K/Akt signalling; see **b,c**). Data, mean±SEM. n=3 livers/condition à 12 pictures each; n=36 canalicular ROIs (**d**). **c,** n=3 assays; unpaired t-tests and 2way ANOVA + Bonferroni's test. **d,** Unpaired t-test (0 vs. 15 min and 30 min, respectively, and 15 min vs. 30 min; n.s.) and 2way ANOVA + Bonferroni's test (stimulated vs. unstimulated at both 15 min and 30 min, respectively; n.s.).

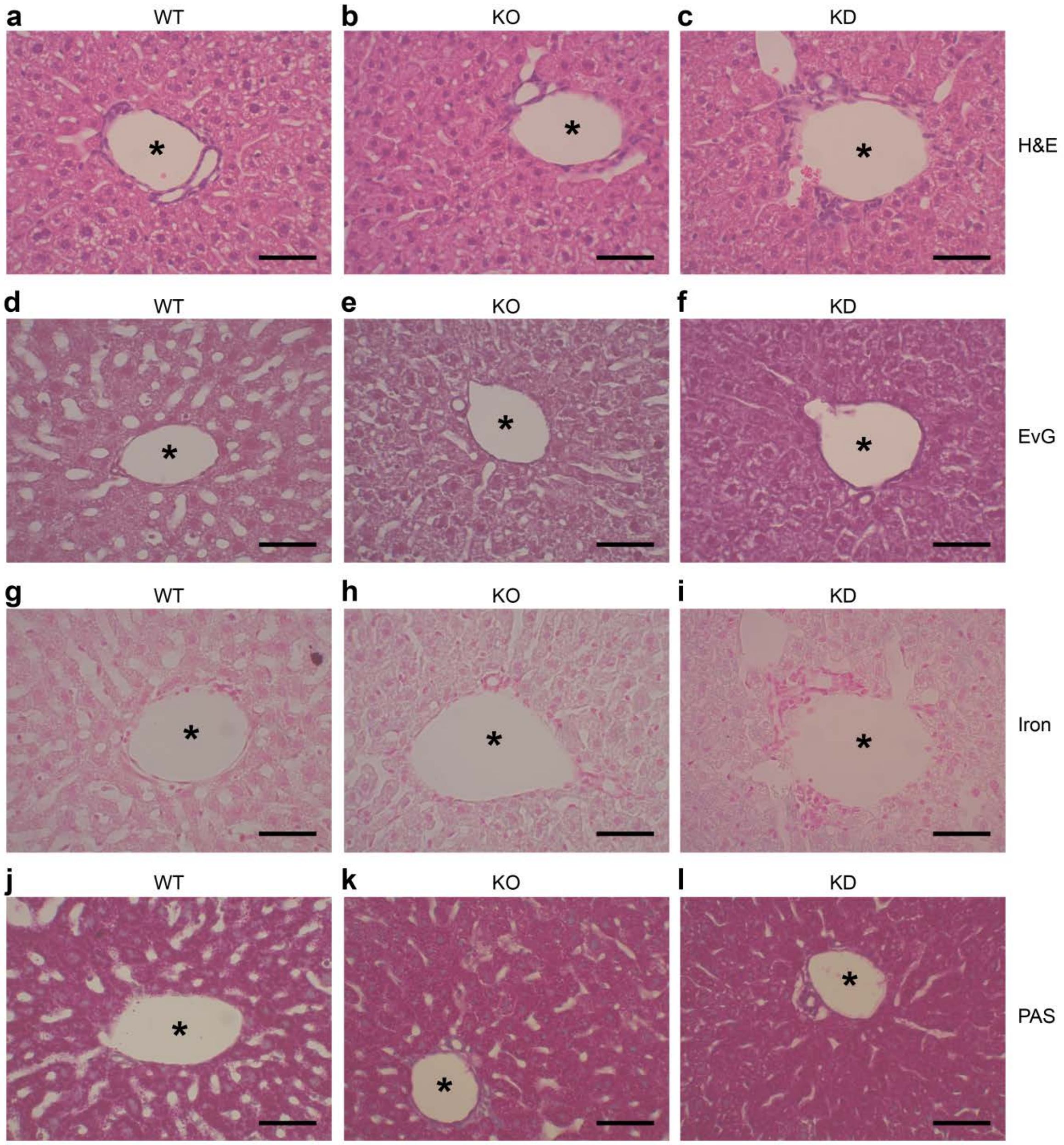

Figure S4. PI3Kγ KO and KD mice do not show any gross alterations of liver morphology and organisation.

Bright field images of liver sections of WT (**a,d,g,j**), PI3Kγ KO (**b,e,h,k**) and PI3Kγ KD (**c,f,i,l**) mice subjected to different histological examinations, H&E **(a-c)**, EvG **(d-f)**, iron (**g-i**) and PAS staining (**j-l**). *, blood vessel. Bars, 250 µm.

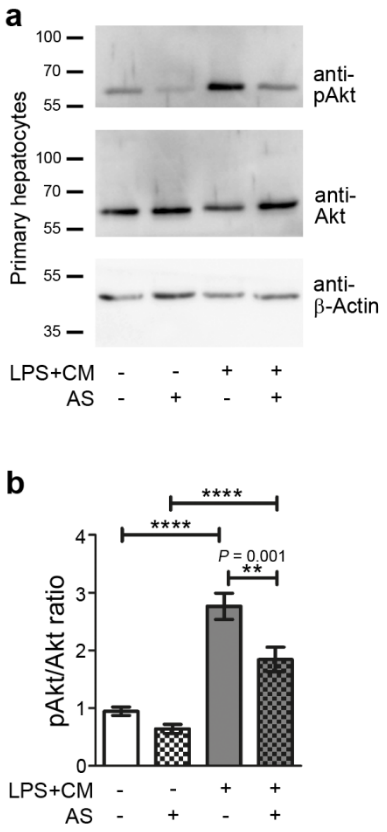

Figure S5. Stimulation of primary hepatocytes with LPS and cytokine mix leads to Akt signalling partially sensitive to the PI3Kγ inhibitor AS605240.

a, Anti-pAkt/Akt immunoblotting analyses of primary hepatocytes isolated from WT mice that were either left untreated (control; -) or stimulated with a combination of LPS and cytokine mix (CM) and either subjected to inhibition with AS605240 (AS) or not. Anti-β-actin immunosignals served as loading control. **b,** Quantitative analyses. Data, mean±SEM. n=8 independent experiments. Statistical analyses, 2way ANOVA + Bonferroni's posttest of stimulated vs. unstimulated (both comparisons $P < 0.0001$) and of inhibited vs. control (comparisons n.s. and $P = 0.001$, respectively). **, $P < 0.01$; ****, $P < 0.0001$. For $P < 0.0001$, exact P values are not available. Other P values are presented in the figure.

Supplementary Information: Uncut images of blots

Figure 1a

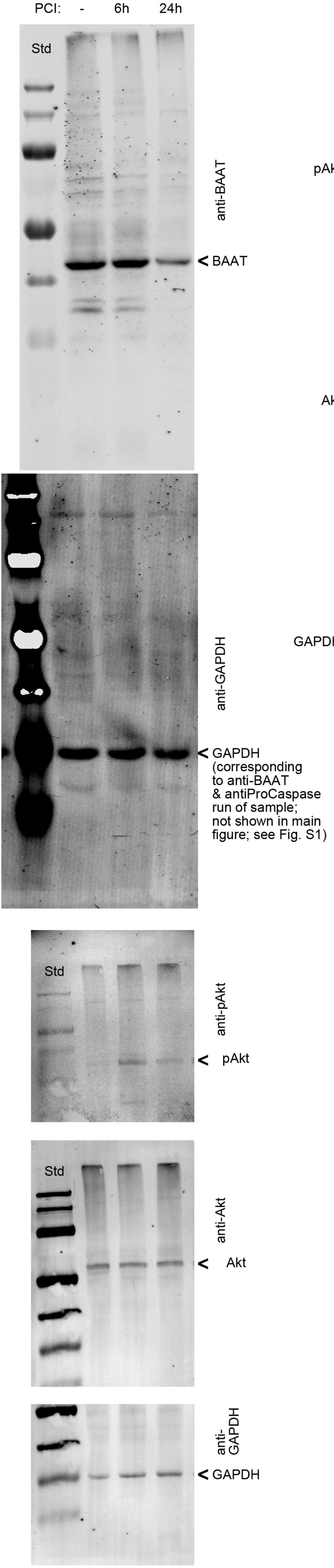

Figure 1c

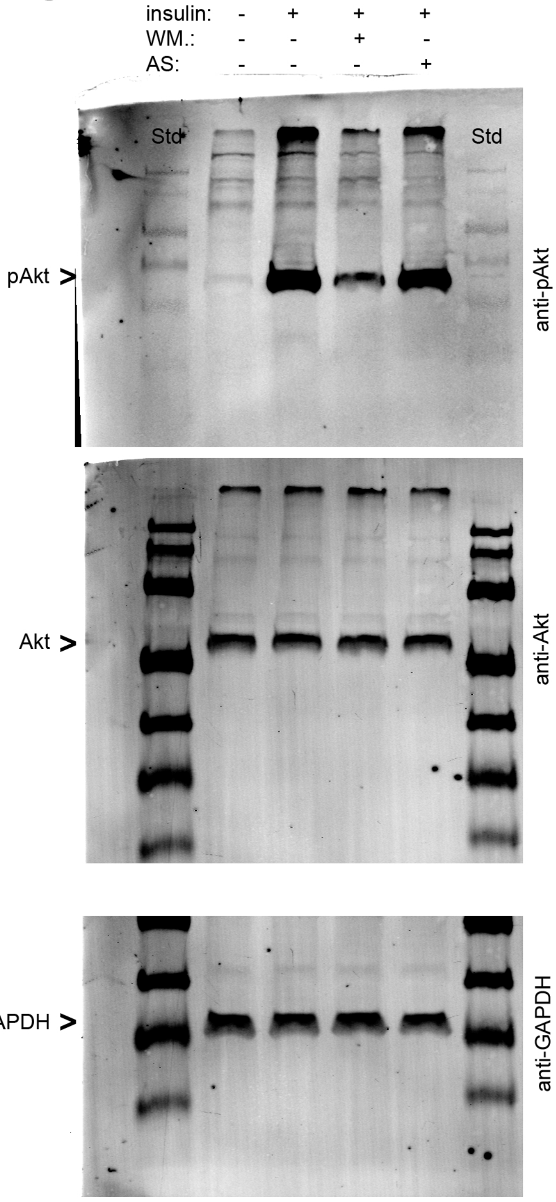

Figure 2a

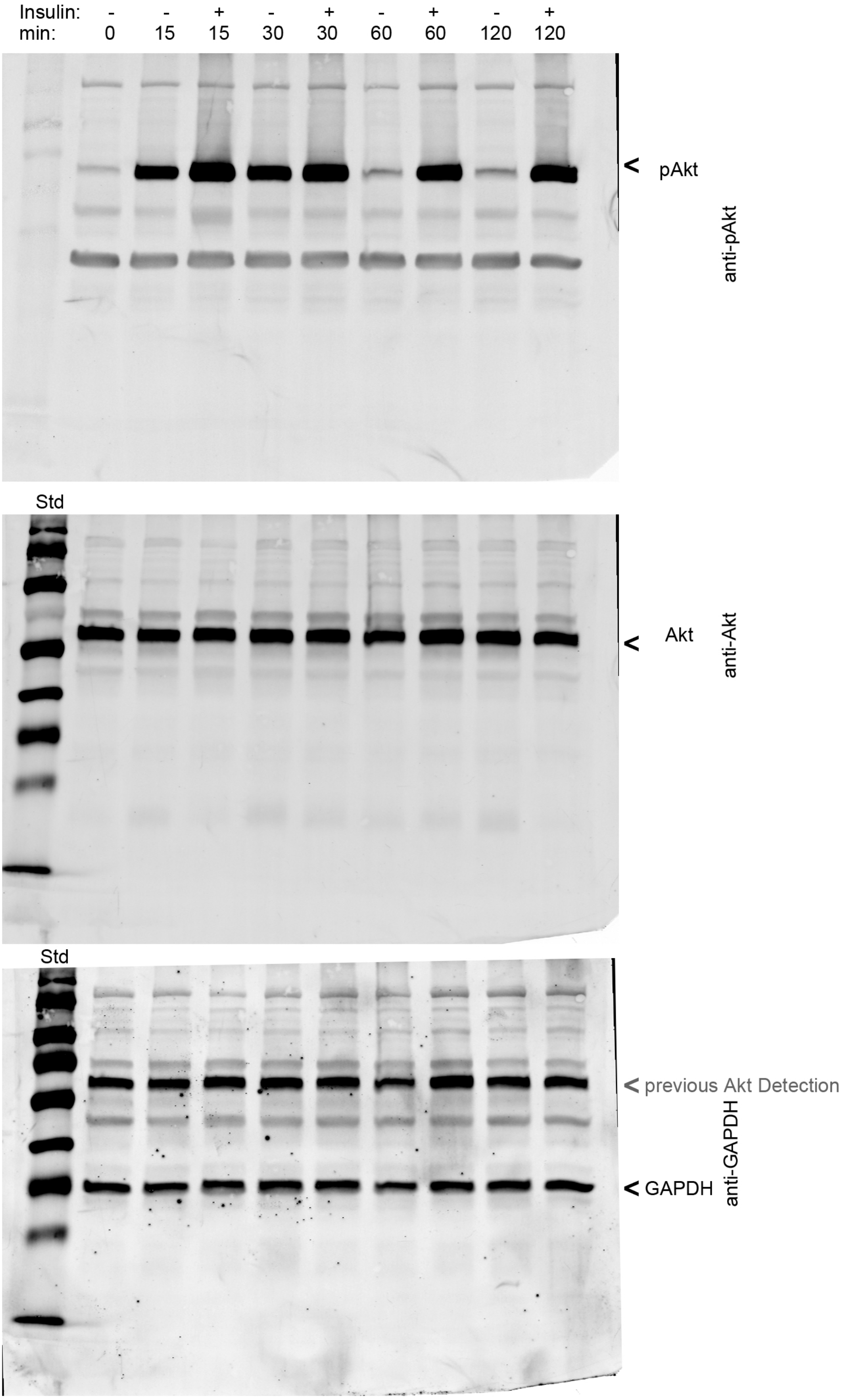
Insulin: - - + - + - + - +
min: 0 15 15 30 30 60 60 120 120
pAkt
anti-pAkt
Std
Akt
anti-Akt
Std
previous Akt Detection
GAPDH
anti-GAPDH

Figure S1

(blot images are shown uncut in the Suppl. Fig. S1)

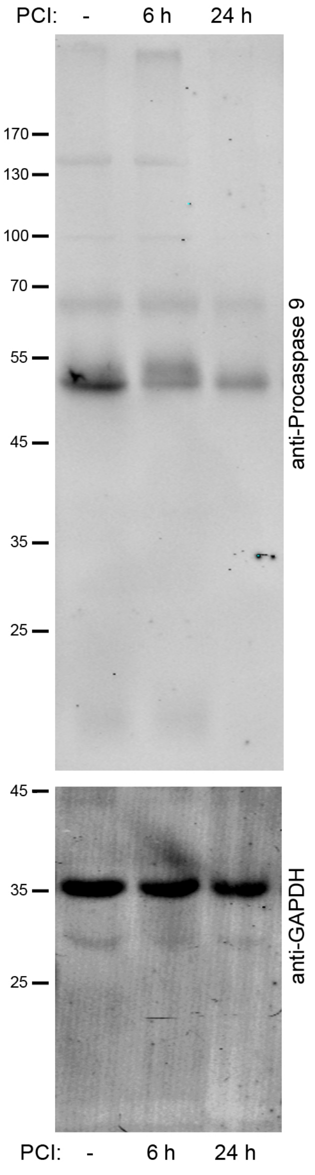

(Blot physically cut; full image)

Figure S2e

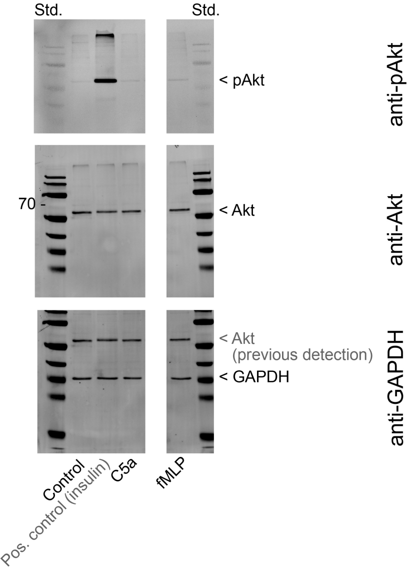
Std.
Std.
< pAkt
anti-pAkt
70 -
< Akt
anti-Akt
< Akt
(previous detection)
< GAPDH
anti-GAPDH
Control
Pos. control (insulin)
C5a
fMLP

Figure S3a

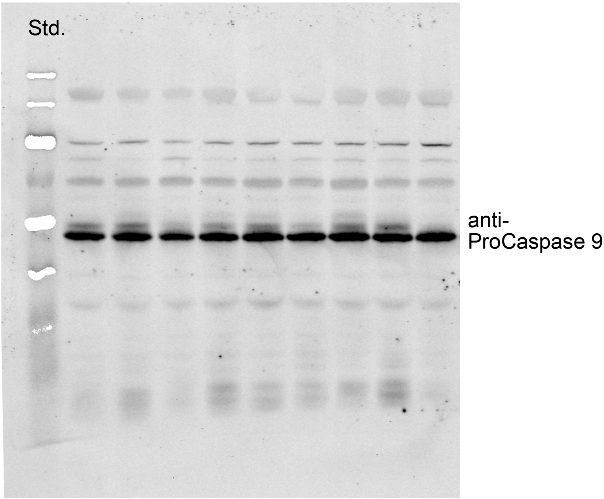

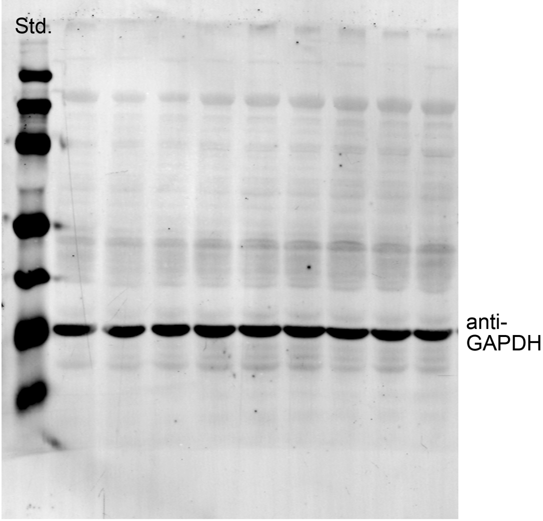

Figure S3b

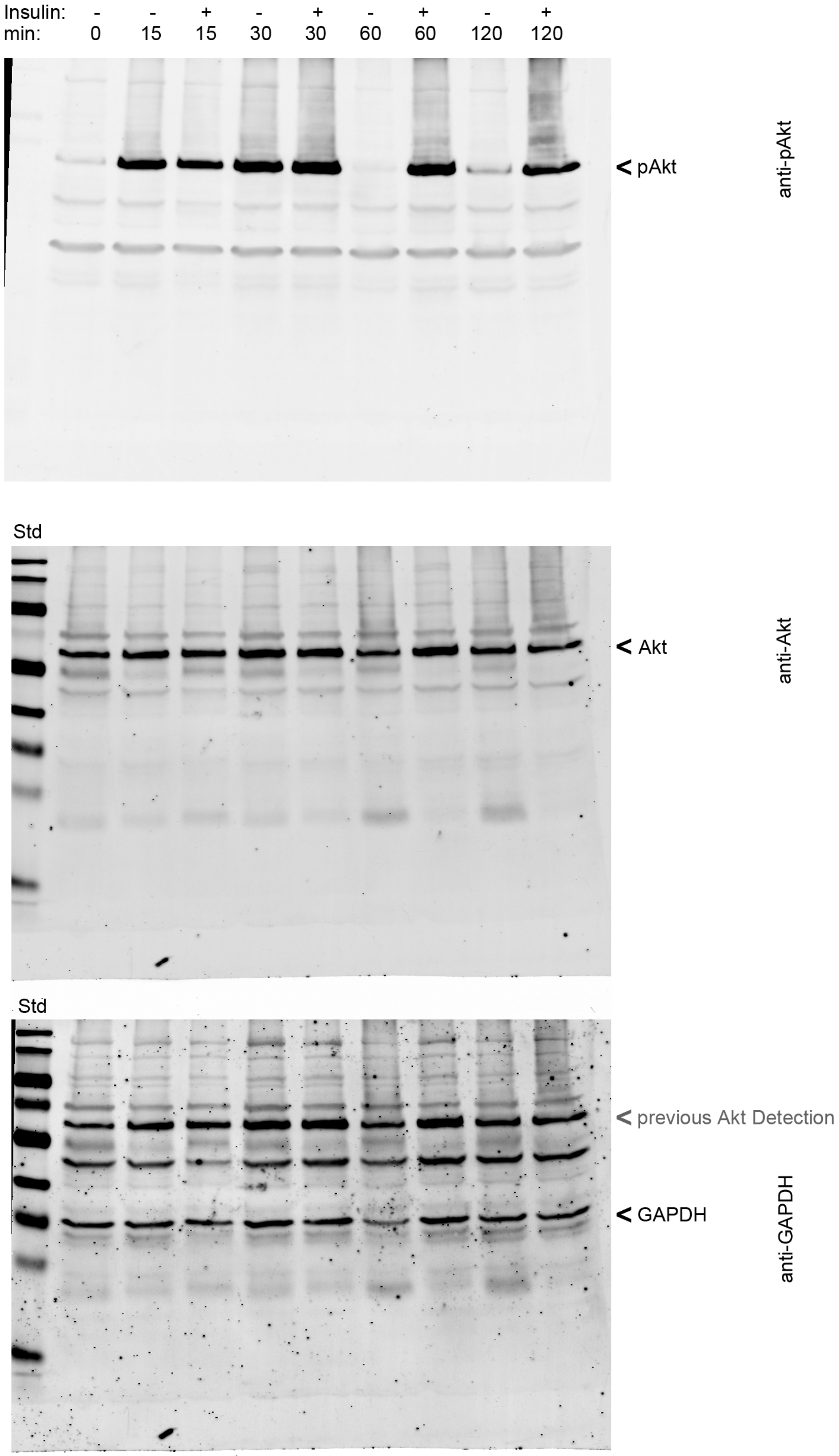
Insulin: - - + - + - + - +
min: 0 15 15 30 30 60 60 120 120
< pAkt
anti-pAkt
Std
< Akt
anti-Akt
Std
< previous Akt Detection
< GAPDH
anti-GAPDH

Figure S5a

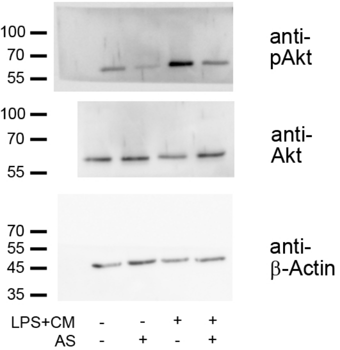
100
70
55
anti-
pAkt
100
70
55
anti-
Akt
70
55
45
35
anti-
β-Actin
LPS+CM - - + +
AS - + - +
